# Expression status transition of *NOTCH1* accompanies chromatin remodeling in human early retinal progenitor cells

**DOI:** 10.12688/f1000research.159630.2

**Published:** 2025-09-22

**Authors:** Yoshitoku Watabe, Sakurako Kobayashi, Takahiro Nakayama, Satoru Takahashi, Masaharu Yoshihara

**Affiliations:** 1 College of Medicine, School of Medicine and Health Sciences, University of Tsukuba, Tsukuba, Ibaraki Prefecture, Japan; 2 Department of Anatomy and Embryology, Institute of Medicine, University of Tsukuba, Tsukuba, Japan; 3 Transborder Medical Center, Institute of Medicine, University of Tsukuba, Tsukuba, Japan; 4 Department of Primary Care and Medical Education, Institute of Medicine, University of Tsukuba, Tsukuba, Japan

**Keywords:** differentiation, epigenetics, eye development, single-cell ATAC-seq, single-cell RNA-seq

## Abstract

**Background:**

The regulation of receptor expression is crucial for fine-tuned signal transduction. Notch signaling is a key signaling pathway involved in retinal development. Compared to the knowledge on this signaling pathway in the differentiation of retinal ganglion cells, less is known about its involvement in earlier stages of retinal progenitor cell differentiation and its regulation, although *NOTCH1,* and probably *NOTCH3,* are involved in earlier stage differentiation. We aimed to clarify the timing of Notch receptor expression in undifferentiated retinal progenitor cells and elucidate the possible involvement of chromatin remodeling in the regulation of Notch receptor expressions.

**Methods:**

We re-analyzed publicly available human fetal retina single-cell RNA-seq and ATAC-seq data (GSE183684) using Seurat/Signac pipelines.

**Results:**

On days 59, 74 and 78, we observed *NOTCH1* and *NOTCH3* mRNA expressions in early retinal progenitor cells, which diminished at later stages of differentiation. Integration of single-cell RNA-seq and ATAC-seq revealed that chromatin remodeling in part of the *NOTCH1* and *NOTCH3* loci were accompanied by transitions in their mRNA expressions. Importantly, the *NOTCH1* locus, which showed chromatin remodeling, contained multiple binding motifs for transcription factors important in early retinal progenitor cell differentiation.

**Conclusions:**

These results suggest that chromatin remodeling may be involved in the differential expression of *NOTCH1*, although another type of Notch mRNA expression regulations may also exist.

## Introduction

Signal transduction depends on the expression of receptors regulated at multiple levels. These regulations include chromatin remodeling and DNA-binding proteins, such as transcription factors and transcriptional repressors. Because fine-tuned signal transduction is necessary for development, it is important to clarify the regulatory mechanisms of receptor expression for understanding the regulation of cell differentiation and subsequent tissue development.

Notch signaling in mammals is dependent on the binding of five canonical DSL ligands and four Notch receptors. ^1^ It has been suggested that Notch loci are subject to chromatin remodeling under both normal ^2– 7^ and pathological ^8– 16^ conditions. In terms of developmental biology, the retina is a good model for investigating cellular differentiation involving Notch signaling. ^17^ The developed retina is composed of multiple cell types, including retinal ganglion cells, bipolar cells, photoreceptors, amacrine cells, horizontal cells, and Müllar glial cells, all of which originate from the retinal progenitor cells (RPCs). RPC is characterized by the expression of *Lhx2*, *Pax6*, *Rax* and *Vsx2* while there are some additional marker genes for developing horizontal cells/retinal ganglion cells ( *Onecut1*/ *2*), ^18^ developing amacrine cells ( *Elavl*2/4), ^19^ developing photoreceptors/amacrine cells/Müllar glial cells ( *Eef1a1*), ^20^ developing retinal ganglion cells ( *Meis2*), ^21^ developing horizontal cells ( *Lhx1*, *Ptf1a*), ^22^ and glial cells ( *Pax2*). ^23^ Of the four Notch receptors in mammals, *Notch1* has been suggested to be involved in the maintenance of RPCs and differentiation into retinal ganglion cells. ^24^ In addition to *Notch1*, *Notch3* expression has also been suggested in the early RPCs, which potentially affects the amount and oscillation of total Notch signaling via its susceptibility to the receptor cleavage and subsequent signal transduction. ^25^ However, it is unclear when and how Notch receptor expression is switched on and off in RPCs during early differentiation. Here, we re-analyzed a public multi-omics dataset of single-cell RNA-seq and single-cell ATAC-seq from three human fetal retinas ^26^ to clarify the timing of Notch receptor expression and examine the involvement of chromatin remodeling in this receptor expression switch.

## Methods

A single-cell multi-omics dataset (GSE183684) ^26^ was downloaded from the Gene Expression Omnibus ( https://www.ncbi.nlm.nih.gov/geo/); data from days 59, 74 and 78 in the dataset were selectively used because they contained many undifferentiated retinal progenitor cells. The data were processed in Seurat version 5.1.0 ^27^ and Signac version 1.14.0 ^28^ pipelines in R version 4.4.1 on the Ubuntu 22.04.4 LTS environment. Pseudotime analysis and integration of the single-cell RNA-seq data and the single-cell ATAC-seq data was conducted by using the “FindTransferAnchors” function of Signac and Monocle3 version 1.3.7, ^29^ respectively. The conditions used in the analysis are provided in the GitHub repository. We searched the transcription factor binding motifs for LHX2 (TAATTA), ^30^ PAX6 (CGCCTGA), ^31^^,^^32^ RAX ([C/T]AATTA) ^33^^,^^34^ and VSX2 (TAATT [A/G]) ^35^ (“Supplementary_table_motif.xlsx”) manually by referencing human genome assembly hg38 in the University of California Santa Cruz genome browser ( https://genome.ucsc.edu/).

## Results

### Re-analysis day 59 human fetal retina

First, early gestational stage samples were characterized (day 59). Uniform manifold approximation and projection (UMAP) analysis of single-cell RNA-seq identified 14 clusters ( Figure 1A), which were further characterized by marker gene expressions ( Figure 1B). This sample primarily contained RPCs with various differentiation statuses except for *PAX2*-expressing glial cells. We defined *LHX2*, *PAX6*, *RAX* and *VSX2* as early markers. We considered that early markers-expressing RPCs and *MKI67*-expressing RPCs constitute early RPCs. In addition, pseudotime analysis suggested that RPCs decreased expressions of *LHX2*, *PAX6*, *RAX* and *VSX2* during differentiation into either *ONECUT1*/ *2*-, *ELAVL2*/ *4*- or *EEF1A1*-expressing RPCs ( Figure 1C). The details of each cluster are available in “Supplementary_table_count.xlsx” in our GitHub repository. Next, we examined Notch mRNA expressions and found that *NOTCH1*, *NOTCH2* and *NOTCH3* were expressed primarily in early RPCs with *NOTCH1* showing the highest expression, which prompted us to focus on *NOTCH1* and *NOTCH3*, ^25^ whereas *NOTCH4* expression was barely detectable ( Figure 1D). Then, we re-analyzed single-cell ATAC-seq data, which was mathematically integrated with its single-cell RNA-seq data by using the “FindTransferAnchors” function of Signac ( Figure 1E). In the early RPCs (Early markers-expressing RPC1- 3_d59 and *MKI67*-expressing RPC1- 2_d59), we observed multiple peaks in the 100 kb upstream and downstream chromosome regions of *NOTCH1*, some of which were diminished in the other RPC clusters such as *ELAVL2*/ *4*- or *EEF1A1*-expressing RPCs ( Figure 2A, arrowheads). Since these regions contained multiple binding motifs for LHX2, PAX6, RAX and VSX2 ( Figure 2A, arrowheads numbered 1-3) (“Supplementary_table_motif.xlsx”), chromatin remodeling of these regions possibly regulates *NOTCH1* expression transition during RPC differentiation although the expression transitions of *LHX2*, *PAX6*, *RAX* and *VSX2* may also support the *NOTCH1* expression transition ( Figure 2A). Since *NOTCH3* has also been suggested for early RPC differentiation, ^25^ we also conducted chromatin accessibility analysis of *NOTCH3* and observed less prominent chromatin remodeling in the 100 kb upstream and downstream regions spanning the *NOTCH3* locus and the mRNA diminishment supports the previous report ^25^ ( Figure 2B). The tables of the chromosome regions of each variable peak in *NOTCH1* and *NOTCH3* are provided in our GitHub repository as “ATAC_peaks_NOTCH1_d59.csv” and “ATAC_peaks_NOTCH3_d59.csv”, respectively. Although *NOTCH2* and *NOTCH4* have been less documented for RPC differentiation in the literature, we have provided the dot plots of the Notch expressions and the coverage plots of *NOTCH2* and *NOTCH4* in “Additional_file_1.tif” in our GitHub repository. In contrast to *NOTCH2* mRNA expression in the early RPCs ( Figure 1D), we observed several peaks in the chromosome region spanning the *NOTCH2* locus although it was difficult to point out the peak regions specific for the early RPCs (Additional_file_1C). Conversely, in contrast to little *NOTCH4* mRNA expression ( Figure 1D), we found that multiple peaks were constantly open in the *NOTCH4* locus during RPC differentiation (Additional_file_1D, arrowheads), some of which contained multiple binding motifs for LHX2, PAX6, RAX and VSX2 (Additional_file_1D, arrowheads numbered 1-2) (“Supplementary_table_motif.xlsx”). The table of the chromosome regions of each variable peak in *NOTCH4* is provided in our GitHub repository as “ATAC_peaks_NOTCH4_d59.csv.”

**Figure 1.**
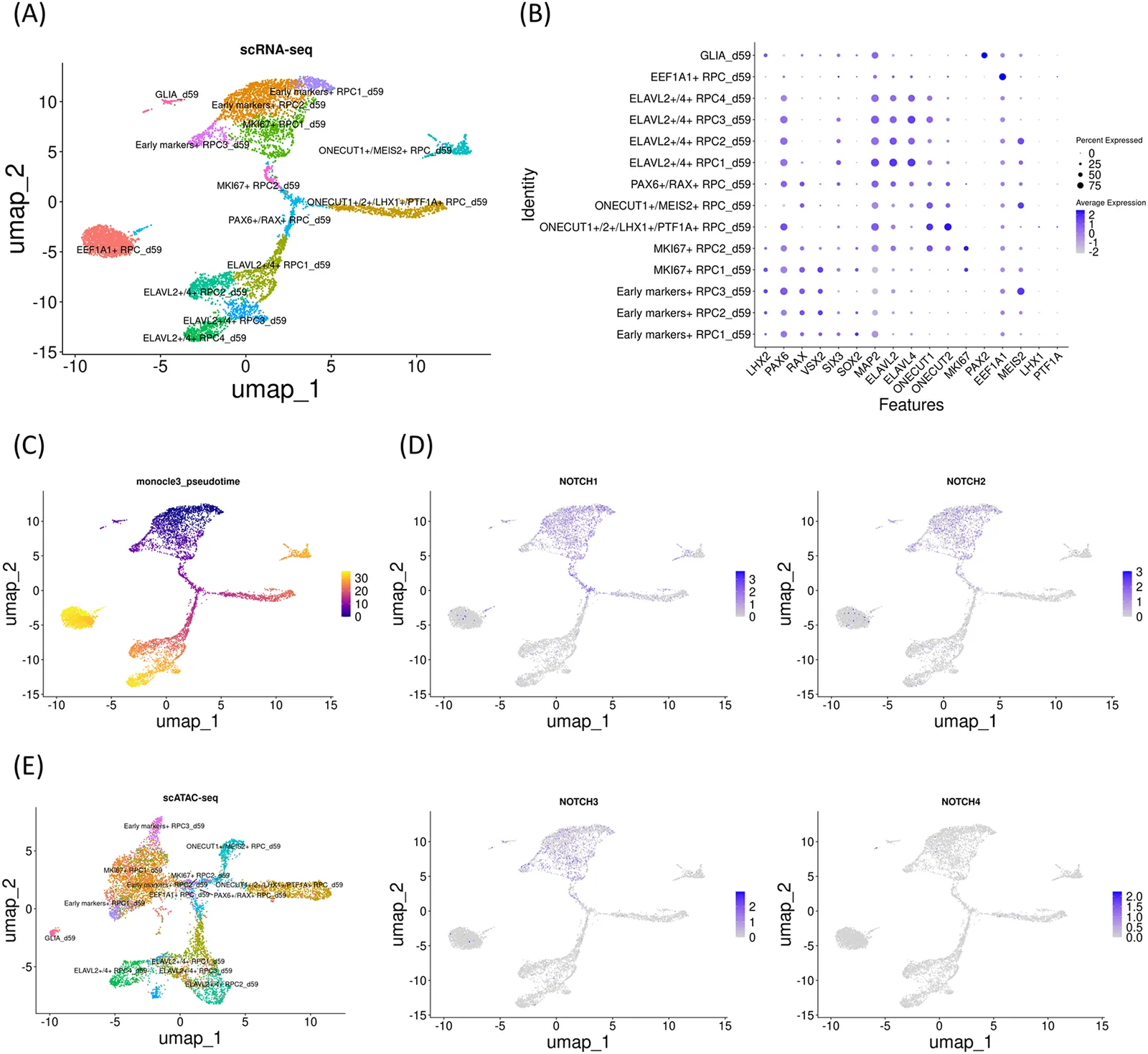
Notch mRNA expression decrease in the day 59 sample. UMAP analysis of the single-cell RNA-seq data identified 14 clusters. (B) Dot plot of marker genes. (C) Monocle3 pseudotime analysis for clarifying the differentiation status. (D) Feature plot of *NOTCH1*- *4.* Note that *NOTCH1, NOTCH2* and *NOTCH3* expressions were prominent in the early RPC clusters. (E) UMAP analysis of the single-cell ATAC-seq data, which was integrated with the single-cell RNA-seq data.

**Figure 2.**
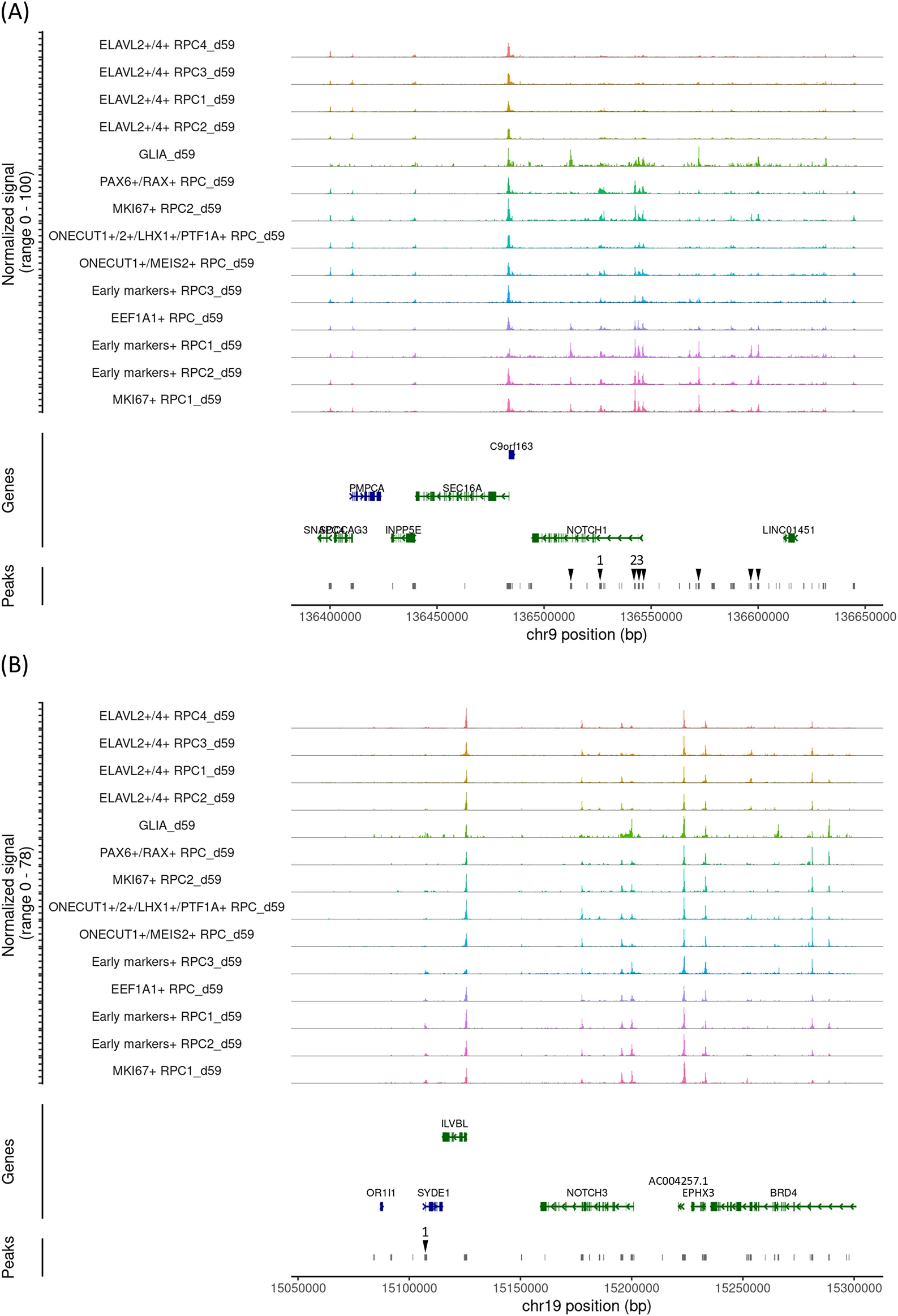
Concomitant chromatin remodeling of *NOTCH1* and *NOTCH3* in the day 59 sample. The coverage plot of the 100 kb upstream and downstream chromosome regions of *NOTCH1*. The upstream of the genes is on the right. Variable peak regions are indicated by arrowheads, with the regions containing transcription binding motifs numbered as 1-3. (B) The coverage plot of the 100 kb upstream and downstream chromosome regions of *NOTCH3*. The upstream of the genes is on the right. A variable peak region which contains transcription binding motifs is indicated by an arrowhead numbered as 1.

### Re-analysis day 74 human fetal retina

To validate the changes in Notch mRNA expressions and chromatin accessibility in the Notch loci during RPC differentiation, we investigated another early gestational stage sample (day 74). UMAP analysis identified 15 clusters ( Figure 3A) that were further characterized by marker gene expressions ( Figure 3B). This sample contained RPCs with various differentiation statuses. Similar to day 59 sample, pseudotime analysis suggested that the early RPCs differentiated into either *ELAVL2*/ *4*, *ONECUT1*/ *MEIS2* or *ONECUT1/2*-expressing RPCs ( Figure 3C). *ONECUT1*/ *MEIS2*-expressing RPCs differentiated into RPCs that markedly expressed *VSX2.* Next, we examined Notch mRNA expressions and found that *NOTCH1*, *NOTCH2* and *NOTCH3* were expressed primarily in early RPCs with *NOTCH1* showing the highest expression whereas *NOTCH4* expression was barely detectable ( Figure 3D), similar to the day 59 sample. We then re-analyzed the single-cell ATAC-seq data, which were integrated with the single-cell RNA-seq data ( Figure 3E). In the early RPCs (Early markers-expressing RPC1- 4_d74 and *MKI67*-expressing RPC_d74), we observed multiple peaks in the 100 kb upstream and downstream chromosome regions of *NOTCH1*, some of which were diminished in the other RPC clusters, such as *ELAVL2*/ *4*- or *ONECUT1/2*-expressing RPCs ( Figure 4A, arrowheads). Since these regions contained multiple binding motifs for LHX2, PAX6, RAX and VSX2 ( Figure 4A, arrowheads numbered 1-4) (“Supplementary_table_motif.xlsx”), chromatin remodeling of these regions possibly regulates *NOTCH1* expression transition during RPC differentiation although the expression transitions of *LHX2*, *PAX6*, *RAX* and *VSX2* may also support the *NOTCH1* expression transition ( Figure 4A). Since *NOTCH3* has also been suggested for early RPC differentiation, ^25^ we also conducted chromatin accessibility analysis of *NOTCH3* and observed less prominent chromatin remodeling in the 100 kb upstream and downstream regions spanning the *NOTCH3* locus ( Figure 4B). The tables of the chromosome regions of each variable peak in *NOTCH1* and *NOTCH3* are provided in our GitHub repository as “ATAC_peaks_NOTCH1_d74.csv” and “ATAC_peaks_NOTCH3_d74.csv”, respectively. In summary, similar to the day 59 sample, in the day 74 sample, a concomitant mRNA decrease and chromatin remodeling in the *NOTCH1* locus were observed. Although *NOTCH2* and *NOTCH4* have been less documented for RPC differentiation in the literature, we have provided the dot plots of the Notch expressions and the coverage plots of *NOTCH2* and *NOTCH4* in “Additional_file_2.tif” in our GitHub repository. In contrast to *NOTCH2* mRNA expression ( Figure 4D), we observed a single peak in the *NOTCH2* locus for *EEF1A1*-expressing RPC_d74, and therefore, no peak regions in the *NOTCH2* locus specific for the early RPCs (Additional_file_2C). Converesly, in contrast to little *NOTCH4* mRNA expression ( Figure 4D), we found that multiple peaks were constantly open in the *NOTCH4* locus during RPC differentiation (Additional_file_2D, arrowheads), some of which contained multiple binding motifs for LHX2, PAX6, RAX and VSX2 (Additional_file_2D, arrowheads numbered 1-3) (“Supplementary_table_motif.xlsx”). The table of the chromosome regions of each variable peak in *NOTCH4* is provided in our GitHub repository as “ATAC_peaks_NOTCH4_d74.csv.”

**Figure 3.**
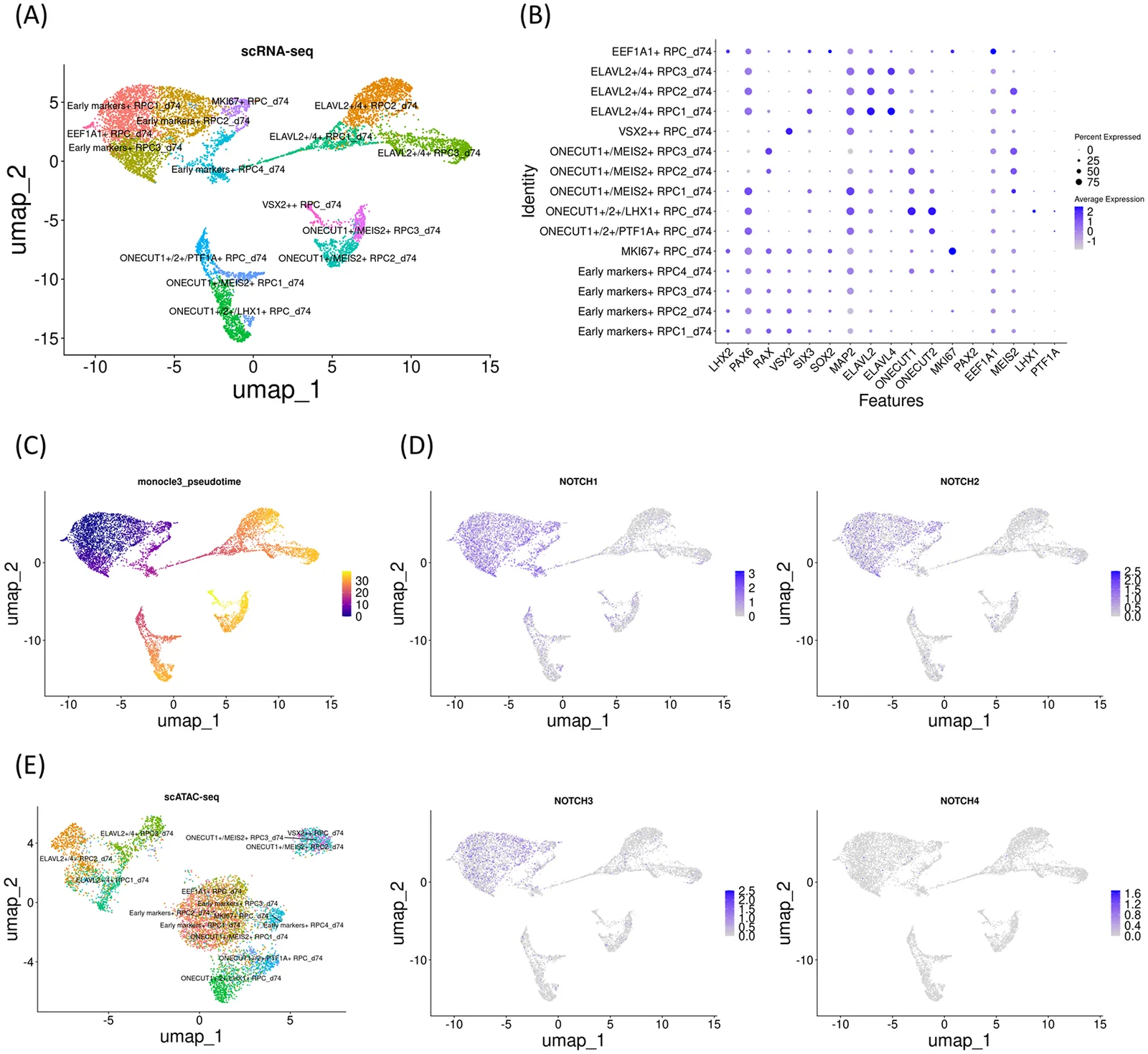
Notch mRNA expression decrease in the day 74 sample. (A) UMAP analysis of the single-cell RNA-seq data identified 15 clusters. (B) Dot plot of marker genes. (C) Monocle3 pseudotime analysis. (D) Feature plot of *NOTCH1*- *4.* Note that *NOTCH1*, *NOTCH2* and *NOTCH3* expressions were prominent in the early RPC clusters. (E) UMAP analysis of the single-cell ATAC-seq data which was integrated with the single-cell RNA-seq data.

**Figure 4.**
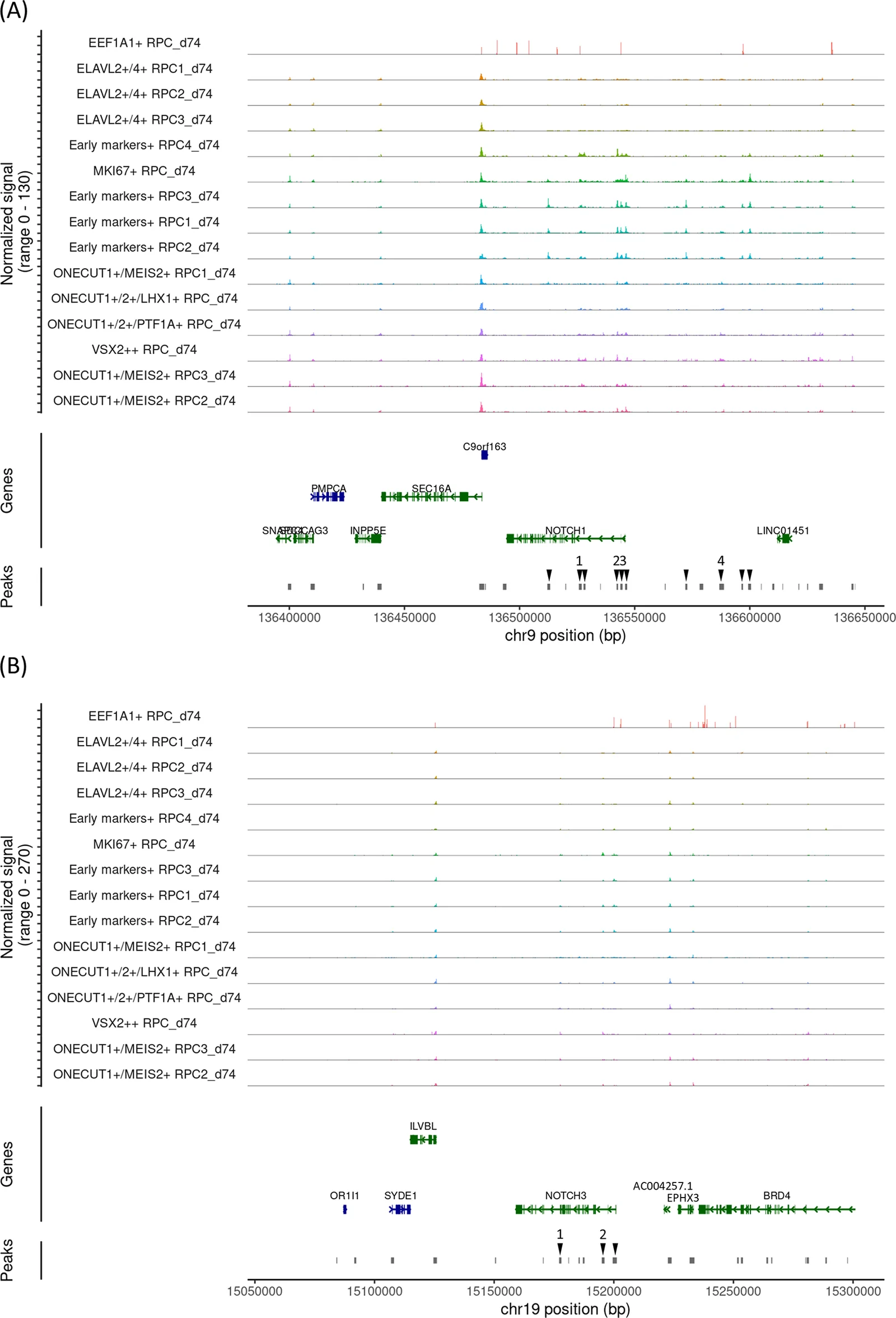
Concomitant chromatin remodeling of *NOTCH1* and *NOTCH3* in the day 74 sample. (A) The coverage plot of the 100 kb upstream and downstream chromosome regions of *NOTCH1*. The upstream of the gene is on the right. Variable peak regions are indicated by arrowheads, with the regions containing transcription binding motifs numbered as 1-4. (B) The coverage plot of the 100 kb upstream and downstream chromosome regions of *NOTCH3*. The upstream of the gene is on the right. Variable peak regions are indicated by arrowheads, with the regions containing transcription binding motifs numbered as 1-2.

### Re-analysis day 78 human fetal retina

To further confirm the developmental changes in Notch mRNA expression and chromatin accessibility at the Notch loci, we added one more early gestational stage sample (day 78). UMAP analysis identified 18 clusters ( Figure 5A) that were further characterized by marker gene expressions ( Figure 5B). The sample contained RPCs with various differentiation statuses. Pseudotime analysis suggested that the early RPCs differentiated into either *ONECUT1*/ *2*-expressing RPCs, which later differentiated into *PTF1A* and *LHX1*-expressing RPCs, *ONECUT1*/ *MEIS2*-expressing RPCs, or *ELAVL2/4*-expressing RPCs ( Figure 5C). Next, we examined Notch mRNA expressions and found that *NOTCH1*, *NOTCH2* and *NOTCH3* were expressed primarily in early RPCs with *NOTCH1* showing the highest expression whereas *NOTCH4* expression was barely detectable ( Figure 5D), similar to the day 59 and 74 samples. We then re-analyzed the single-cell ATAC-seq data, which was integrated with the single-cell RNA-seq data ( Figure 5E). In the early RPCs (Early markers-expressing RPC1-3_d78 and *MKI67*-expressing RPC1-2_78), we observed multiple peaks in the 100 kb upstream and downstream chromosome regions of *NOTCH1*, some of which were diminished in the other RPC clusters ( Figure 6A, arrowheads). Since these regions contained multiple binding motifs for LHX2, PAX6, RAX and VSX2 ( Figure 6A, arrowheads numbered 1-4) (“Supplementary_table_motif.xlsx”), chromatin remodeling of these regions possibly regulates *NOTCH1* expression transition during RPC differentiation although the expression transitions of *LHX2*, *PAX6*, *RAX* and *VSX2* may also support the *NOTCH1* expression transition ( Figure 6A). Since *NOTCH3* has also been suggested for early RPC differentiation, ^25^ we also conducted chromatin accessibility analysis of *NOTCH3* and observed less prominent chromatin remodeling in the 100 kb upstream and downstream regions spanning the *NOTCH3* locus ( Figure 6B). The tables of the chromosome regions of each variable peak in *NOTCH1* and *NOTCH3* are provided in our GitHub repository as “ATAC_peaks_NOTCH1_d78.csv” and “ATAC_peaks_NOTCH3_d78.csv”, respectively. In summary, examinations of all the three independent samples suggested that *NOTCH1* mRNA expression decreased as RPC differentiation progresses, which was concomitant with chromatin remodeling in the *NOTCH1* locus. Importantly, chromatin remodeling regions contained multiple transcription factor binding motifs. Although *NOTCH2* and *NOTCH4* have been less documented for RPC differentiation in the literature, we have provided the dot plots of the Notch expressions and the coverage plots of *NOTCH2* and *NOTCH4* in “Additional_file_3.tif” in our GitHub repository. In contrast to *NOTCH2* mRNA expression ( Figure 5D), we observed several peaks in the chromosome regions spanning the *NOTCH2* locus although it was difficult to point out peak regions specific for the early RPCs (Additional_file_3C). Conversly, in contrast to little *NOTCH4* mRNA expression ( Figure 5D), we found that multiple peaks were constantly open in the *NOTCH4* locus during RPC differentiation (Additional_file_3D, arrowheads), some of which contained multiple binding motifs for LHX2, PAX6, RAX and VSX2 (Additional_file_3D, arrowheads numbered 1-3) (“Supplementary_table_motif.xlsx”). The table of the chromosome regions of each variable peak in *NOTCH4* is provided in our GitHub repository as “ATAC_peaks_NOTCH4_d78.csv.”

**Figure 5.**
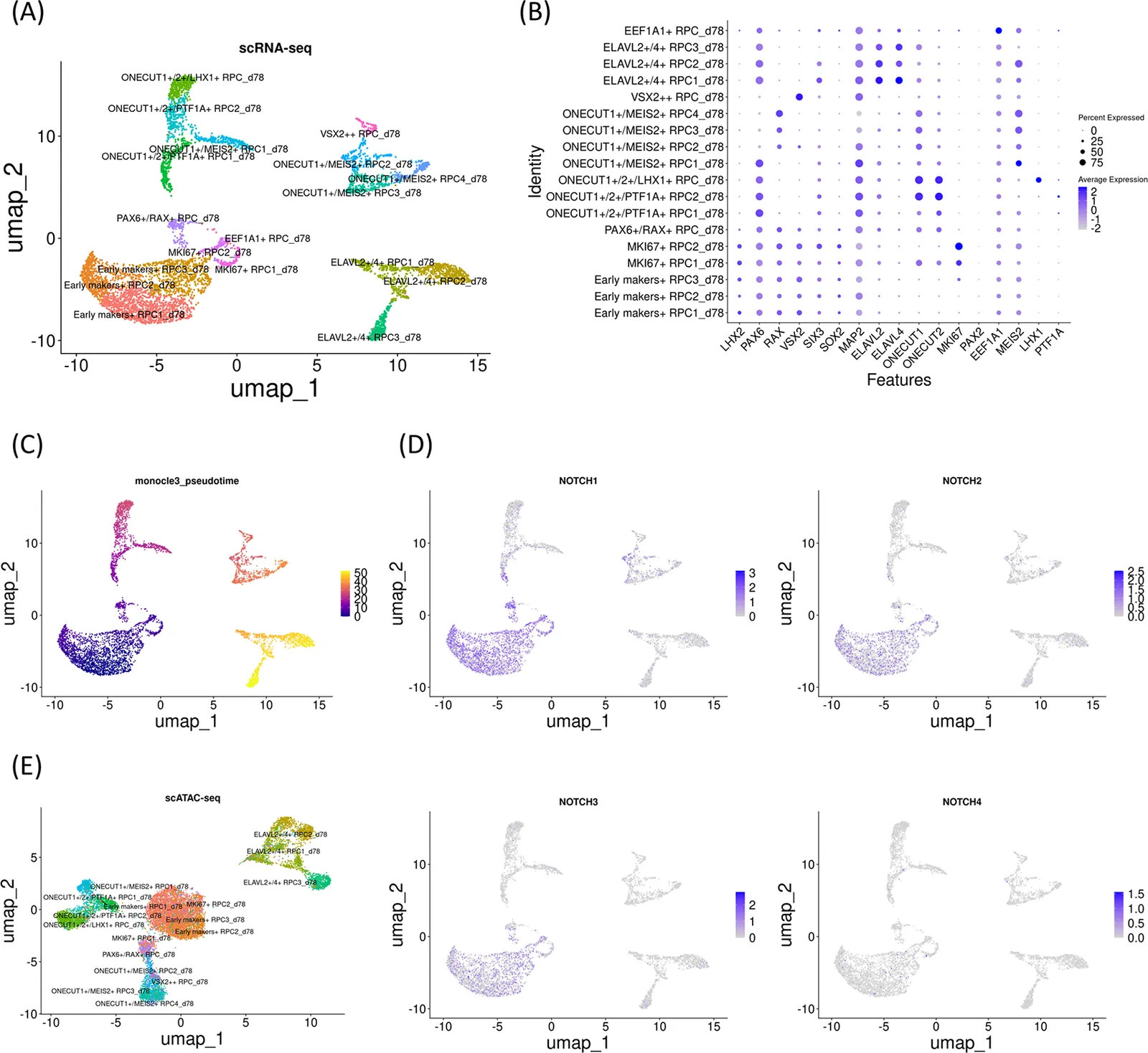
Notch mRNA expression decrease in the day 78 sample. (A) UMAP analysis of the single-cell RNA-seq data identified 18 clusters. (B) Dot plot of marker genes. (C) Monocle3 pseudotime analysis. (D) Feature plot of *NOTCH1- 4*. Note that *NOTCH1*, *NOTCH2* and *NOTCH3* expressions were prominent in early RPC clusters. (E) UMAP analysis of the single-cell ATAC-seq data which was integrated with the single-cell RNA-seq data.

**Figure 6.**
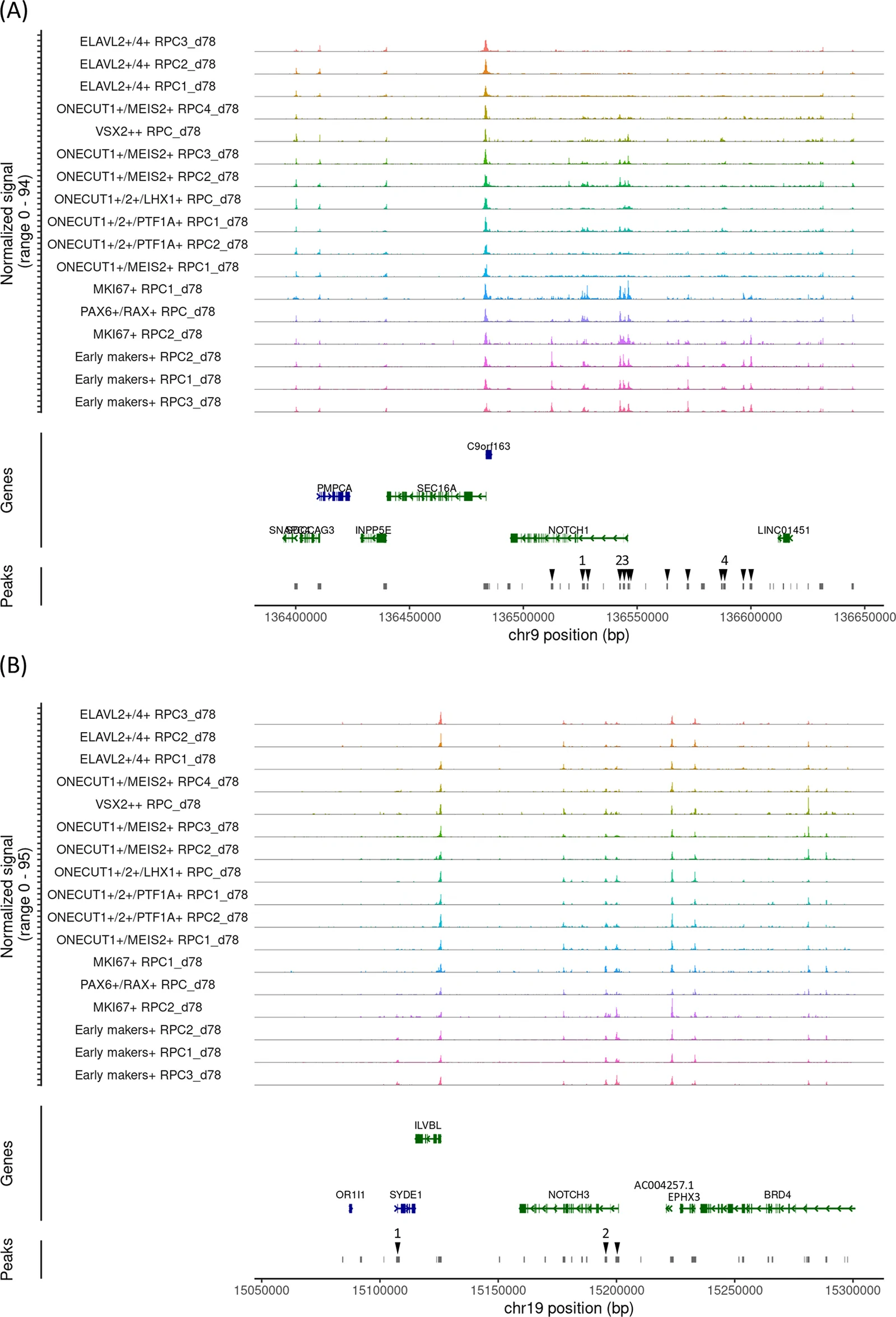
Concomitant chromatin remodeling of *NOTCH1* and *NOTCH3* in the day 78 sample. (A) The coverage plot of the 100 kb upstream and downstream chromosome regions of *NOTCH1*. The upstream of the gene is on the right. Variable peak regions are indicated by arrowheads, with the regions containing transcription binding motifs numbered as 1-4. (B) The coverage plot of the 100 kb upstream and downstream chromosome regions of *NOTCH3*. The upstream of the gene is on the right. Variable peak regions are indicated by arrowheads, with the regions with transcription binding motifs are numbered as 1-2.

## Discussion

The involvement of Notch signaling in cell fate choices is well documented, including in *Drosophila* neurogenesis ^36^ and mammalian biliary development. ^37^ Although the regulation of Notch receptor expression is necessary for these processes, to the best of our knowledge, few studies have used genome-wide investigations of the underlying molecular mechanisms. To examine chromatin remodeling in such regulatory mechanisms, we re-analyzed a single-cell RNA-seq and ATAC-seq dataset from developing retinas in which differentiation trajectories were well characterized. By re-analyzing three independent samples, we observed chromatin remodeling in the *NOTCH1* locus, which contains multiple binding motifs for transcription factors, concomitant with changes in its mRNA expression during RPC differentiation. These findings highlight that the fine-tuned regulation of Notch receptor expressions occurs at an epigenetic level.

*NOTCH1* and *NOTCH3* were well documented in the early RPC differentiation compared to *NOTCH2* and *NOTCH4*. Indeed, we observed *NOTCH1* and *NOTCH3* mRNA expressions in contrast to little *NOTCH4* mRNA expression throughout the three samples. We observed comparable *NOTCH2* mRNA expression in the early RPCs although we are unable to fully explain the discrepancy between mRNA expression and chromatin accessibility regarding this gene owing to lack of significant peak regions in the *NOTCH2* locus (Additional_file_1C, 2C and 3C). Some possible causes such as regulation via distal enhancer and technical limitations of ATAC-seq may exist. When *NOTCH2* mRNA is transcribed in support with those mechanisms, the decrease of *NOTCH2* mRNA expression during RPC differentiation may be influenced by a post-transcriptional mechanism since a small non-coding regulatory RNA (CAT1) reportedly promotes stabilization of *Notch2* mRNA. ^38^ The roles and regulations of *NOTCH2* are, however, beyond the scope of this study. Conversely, we observed little *NOTCH4* mRNA expression and its high chromatin accessibility with multiple binding motifs for LHX2, PAX6, RAX and VSX2. This observation might be owing to the absence of other transcription factors for *NOTCH4* mRNA expression since this gene expression has been suggested to highly depend on the cell type and its expressing transcription factors. For example, *Notch4* is limitedly expressed in endothelial cells, compared to *Notch1*, under regulation of AP-1 transcription factor. ^39^ Although we are unable to provide solid conclusions on the expression and regulation of *NOTCH2* and *NOTCH4* in the early RPCs, we believe that this does not impede our findings that *NOTCH1* mRNA expression transition during RPC differentiation accompanied chromatin remodeling.

An ophthalmological study revealed that the epigenetic landscape of cell type-specific enhancers shifted during differentiation of RPCs. ^40^ For example, in the single-cell ATAC-seq data from embryonic day 14.5 mouse retina, motif enrichment for *Lhx2*, *Rax* and *Pax6* in the early RPCs were observed, and footprinting analysis validated binding of those transcription factors to their motifs. These high chromatin accessibilities decreased as they differentiated into retinal ganglion cells and non-retinal ganglion cells. Although that study is excellent in providing comprehensive and in-depth insights, ours is unique in focusing on the Notch loci for clarifying the regulatory mechanisms in view of Notch signaling biology.

Finally, we note that further investigations, such as a large deletion of these regions, will be needed to evaluate the contribution of the identified chromatin remodeling to the differential expression of Notch receptors in RPC subsets.

### Ethical considerations

This study does not generate new data from human. Therefore, we consider that there are no special requirements on recruitment and publication. In addition, this study does not involve analysis of animals and plants.

### Consent for publication

Not applicable.

## Authors’ contributions

Y. W.: Data Curation, Formal Analysis, Software, Visualization, Writing – Original Draft Preparation

S.K.: Writing – Original Draft Preparation

T.N.: Writing – Original Draft Preparation

S.T.: Supervision, Writing – Review & Editing

M.Y.: Conceptualization, Formal Analysis, Funding Acquisition, Methodology, Project Administration, Software, Writing – Original Draft Preparation

## Data Availability

A single-cell multiomics dataset (GSE183684) ^26^ was downloaded from the Gene Expression Omnibus database ( https://www.ncbi.nlm.nih.gov/geo/query/acc.cgi?acc=GSE183684). The original data in the present study including additional data available from: https://github.com/Yoshitokky/ eyeATAC_NOTCH1_data/tree/main. ^41^ Archived software available from: https://doi.org/10.5281/zenodo.17084024. License: OSI approved open license software is under the terms of the Creative Commons Zero “No rights reserved” data waiver (CC0 1.0 Public domain dedication). The project contains the following underlying data:
• Figure_1A.png • Figure_1B.png • Figure_1C.png • Figure_1D_NOTCH1.png • Figure_1D_NOTCH2.png • Figure_1D_NOTCH3.png • Figure_1D_NOTCH4.png • Figure_1E.png • Figure_2A.png • Figure_2B.png • Figure_3A.png • Figure_3B.png • Figure_3C.png • Figure_3D_NOTCH1.png • Figure_3D_NOTCH2.png • Figure_3D_NOTCH3.png • Figure_3D_NOTCH4.png • Figure_3E.png • Figure_4A.png • Figure_4B.png • Figure_5A.png • Figure_5B.png • Figure_5C.png • Figure_5D_NOTCH1.png • Figure_5D_NOTCH2.png • Figure_5D_NOTCH3.png • Figure_5D_NOTCH4.png • Figure_5E.png • Figure_6A.png • Figure_6B.png • Additional_file_1.tif (Extended data for d59 data). • Additional_file_2.tif (Extended data for d74 data). • Additional_file_3.tif (Extended data for d78 data). • Additional_file_1_A.png • Additional_file_1_B.png • Additional_file_1_C.png • Additional_file_1_D.png • Additional_file_2_A.png • Additional_file_2_B.png • Additional_file_2_C.png • Additional_file_2_D.png • Additional_file_3_A.png • Additional_file_3_B.png • Additional_file_3_C.png • Additional_file_3_D.png • Supplementary_table_count.xlsx • Supplementary_table_motif.xlsx • ATAC_peaks_NOTCH1_d59.csv • ATAC_peaks_NOTCH3_d59.csv • ATAC_peaks_NOTCH4_d59.csv • ATAC_peaks_NOTCH1_d74.csv • ATAC_peaks_NOTCH3_d74.csv • ATAC_peaks_NOTCH4_d74.csv • ATAC_peaks_NOTCH1_d78.csv • ATAC_peaks_NOTCH3_d78.csv • ATAC_peaks_NOTCH4_d78.csv

## Software availability

Source code available from: https://github.com/Yoshitokky/eyeATAC_NOTCH1_software/tree/main. ^42^

Archived software available from: https://doi.org/10.5281/zenodo.17084033.

License: OSI approved open license software is under the terms of the Creative Commons Zero “No rights reserved” data waiver (CC0 1.0 Public domain dedication).

The project contains the following underlying data:

• 250907_retina_chromatin_d59_No0.R (R code script for d59 data followed by No1).
• 250907_retina_chromatin_d59_No1.R (R code script for d59 data followed by No2).
• 250907_retina_chromatin_d59_No2.R (R code script for d59 data followed by No3).
• 250907_retina_chromatin_d59_No3.R (R code script for d59 data followed by No4).
• 250907_retina_chromatin_d59_No4.R (R code script for d59 data).
• 250907_retina_chromatin_d74_No0.R (R code script for d74 data followed by No1).
• 250907_retina_chromatin_d74_No1.R (R code script for d74 data followed by No2).
• 250907_retina_chromatin_d74_No2.R (R code script for d74 data followed by No3).
• 250907_retina_chromatin_d74_No3.R (R code script for d74 data followed by No4).
• 250907_retina_chromatin_d74_No4.R (R code script for d74 data).
• 250907_retina_chromatin_d78_No0.R (R code script for d78 data followed by No1).
• 250907_retina_chromatin_d78_No1.R (R code script for d78 data followed by No2).
• 250907_retina_chromatin_d78_No2.R (R code script for d78 data followed by No3).
• 250907_retina_chromatin_d78_No3.R (R code script for d78 data followed by No4).
• 250907_retina_chromatin_d78_No4.R (R code script for d78 data).
