# Expression status transition of *NOTCH1* accompanies chromatin remodeling in human early retinal progenitor cells

**DOI:** 10.12688/f1000research.159630.4

**Published:** 2026-04-17

**Authors:** Yoshitoku Watabe, Sakurako Kobayashi, Takahiro Nakayama, Satoru Takahashi, Masaharu Yoshihara

**Affiliations:** 1 College of Medicine, School of Medicine and Health Sciences, University of Tsukuba, Tsukuba, Ibaraki Prefecture, Japan; 2 Department of Anatomy and Embryology, Institute of Medicine, University of Tsukuba, Tsukuba, Japan; 3 Transborder Medical Center, Institute of Medicine, University of Tsukuba, Tsukuba, Japan; 4 Department of Primary Care and Medical Education, Institute of Medicine, University of Tsukuba, Tsukuba, Japan

**Keywords:** differentiation, epigenetics, eye development, single-cell ATAC-seq, single-cell RNA-seq

## Abstract

**Background:**

Notch signaling, especially via Notch1 and Notch3 receptors, is involved in normal eye development by maintaining retinal progenitor cell state before their differentiation although comprehensive analysis into the expressions and chromatin landscape of the four Notch receptors is still lacking.

**Methods:**

We re-analyzed publicly available human fetal retina single-cell RNA-seq and ATAC-seq data (GSE183684) at days 59, 74 and 78, using Seurat/Signac/Monocle3 pipelines.

**Results:**

We observed *NOTCH1* and *NOTCH3* mRNA expressions in retinal progenitor cells, which diminished at later stages of differentiation. Integration of single-cell RNA-seq and ATAC-seq revealed that chromatin remodeling in the *NOTCH1* locus and less prominently in the *NOTCH3* locus that were accompanied by the decrease of their mRNA expressions. Importantly, those chromatin remodeling regions contained the binding motifs of PAX6 transcription factor that is essential for retinal progenitor cells. Since PAX6 was expressed even in the later differentiation stages and suggested to bind DNA in some cell populations in and after the retinal progenitor cell stage, chromatin remodeling in PAX6’s binding sites might direct *NOTCH1* and *NOTCH3* expression decrease after the retinal progenitor cell stage.

**Conclusions:**

These results suggest that chromatin remodeling may be involved in the differential expression of *NOTCH1* during retinal progenitor cell differentiation.

## Introduction

Signal transduction depends on the expression of receptors regulated at multiple levels. These regulations include chromatin remodeling and DNA-binding proteins, such as transcription factors and transcriptional repressors. Since fine-tuned signal transduction is necessary for development, it is important to clarify the regulatory mechanisms of receptor expression for understanding the regulation of cell differentiation and subsequent tissue development.

The retina is a good model for investigating cellular differentiation since the cell fate determination is well-documented. The developed retina is composed of multiple cell types, including retinal ganglion cells (RGCs), amacrine cells, photoreceptor and bipolar cell precursors (PR/BC Precursors), horizontal cells, rod photoreceptors, cone photoreceptors and Muller glial cells, all of which originate from the retinal progenitor cells (RPCs). RPC is characterized by the expression of *Lhx2*, ^1^ *Pax6,* ^2^ *Rax* ^3^ and *Vsx2* ^4^ while there are some additional marker genes for developing RGCs ( *Rbpms*), ^5^ developing amacrine cells ( *Gad2*), ^6^ PR/BC precursor cells ( *Gadd45g*), ^7^ developing horizontal cells ( *Lhx1*), ^8^ developing rod photoreceptors ( *Otx2*), ^9^ developing cone photoreceptors ( *Otx2*, and *Prdm1* also known as *Blimp1*) ^9^ ^,^ ^10^ and developing Muller glial cells ( *Slc1a3*). ^11^

Notch signaling involves four Notch receptors that are expressed differently in space. In rat embryonic eyes, *Notch1* was expressed in the posterior side of embryonic days (E) 13.5 and E14.5 retinas, *Notch2* was expressed in the retinal pigment epithelium throughout E12.5 and E14.5, and *Notch3* was expressed throughout the retina from E12.5 to E14.5 with strong expression in the anterior side at E14.5. ^12^ ^,^ ^13^ Further analyses on *Notch2* using a knock-in mouse suggested that *Notch2* was expressed at a slightly detectable level from E9.5 optic vesicle stage in the retinal neuroepithelium that contained RPCs. ^14^ In addition, deletion of *Notch2* in outer ciliary epithelium disrupted ciliary body formation, ^15^ suggesting that this gene acts in the temporal side of the eye. Indeed, the spatial information in the mouse retina at E13.5 determined upstream regulators involved in *Hes1* induction, which acts as a hub for Notch-dependent and -independent inputs in RPCs. ^16^ Notably, *Notch4* was expressed in arterial endothelial cells rather than RPCs and their descendants in the retina. ^17^

Notch signaling is also involved in the differentiation from RPCs to the matured cells in a complex way. Loss-of-function of Notch common co-actors *Rbpj* ^18^ and *Maml* ^19^ increased *Crx*-expressing photoreceptor precursor cells and cone/horizontal cell-restricted RPCs, respectively. More specifically, *Notch1* has been shown to inhibit the rod fate and maintain pluripotency of RPCs. ^20^ ^–^ ^22^ This notion is also supported by a single-cell ATAC-seq and disease-associated 5q14.3 enhancer knockout study. ^7^ In addition, *Notch3*, as well as *Notch1*, was shown to be expressed as early as optic vesicle stage. ^23^ Indeed, *Notch3* knockout mice showed reduced number of RGCs ^24^ although zebrafish *Notch3* was also essential for Muller glial cells. ^25^ These results suggest that *Notch1* and *Notch3* act in RPCs to regulate their differentiation although it is unclear how RPC-dominant expressions of *Notch1* and *Notch3* are guaranteed during RPC differentiation.

A murine ophthalmological study revealed that the epigenetic landscape of cell type-specific enhancers shifted during differentiation of RPCs. ^26^ This report examined sequential and combinatorial interactions of transcription factors (TFs) and transcription factor binding sites (TFBSs) during RPC differentiation. Concretely, single-cell ATAC-seq analysis of this study identified chromatin remodeling at RPC-specific enhancers for key TFs including Vsx2 and showed TF-TFBS binding of key transcription factors including Rax, Lhx2 and Pax6 in naïve RPCs. In addition, this study also performed CUT&Tag for Atoh7 and Pou4f2 and identified five Atoh7-binding motifs in the *Notch1* locus. Although this study provides a comprehensive chromatin landscape during RPC differentiation and detailed information focusing on Atoh7 and Pou4f2, ^7^ a comprehensive analysis of Notch signaling in this context is still lacking except for the finding of decrease of *NOTCH1* chromatin accessibility.

The Notch loci are subject to epigenetic regulation under both normal ^27^ ^–^ ^32^ and pathological ^33^ ^–^ ^41^ conditions in a context-dependent manner. To examine the chromatin remodeling in the four Notch loci during retinal development in addition to confirming decrease of *NOTCH1* chromatin accessibility, ^7^ we re-analyzed a publicly available multi-omics dataset of single-cell RNA-seq and single-cell ATAC-seq of human fetal retinas. ^7^

## Methods

A single-cell multi-omics dataset (GSE183684) ^7^ was downloaded from the Gene Expression Omnibus ( https://www.ncbi.nlm.nih.gov/geo/). Data from the earliest three days (day 59, 74 and 78) in this dataset were used to balance computational burden and the number of RPCs. The data were processed in the pipelines using Seurat version 5.4.0, ^42^ Signac version 1.14.0 and 1.16.0, ^43^ and Monocle3 version 1.3.7 ^44^ in R version 4.4.1, 4.5.2 and 4.5.3 due to version updates during the analysis. Detailed session information for each step is provided in the accompanying code. Integration of the single-cell RNA-seq data and the single-cell ATAC-seq data was conducted using the “FindTransferAnchors” function of Signac. We used FIMO tool ^45^ in the MEME Suite ^46^ referencing the JASPAR motif database ( https://jaspar.elixir.no/) and human genome assembly hg38 in the University of California Santa Cruz genome browser ( https://genome.ucsc.edu/) to search transcription factor binding motifs. We also conducted footprinting analysis in Signac pipeline. The R codes used in the analysis are provided in our GitHub repository with archive in Zenodo.

## Results

We used “RunHarmony” function ^47^ with the Seurat pipeline to reduce batch effect when we integrated and re-analyzed the single-cell RNA-seq data of human fetal retinas at day 59, 74 and 78. These samples were well-overlapped in the uniform manifold approximation and projection (UMAP) analysis ( Figure 1A). We annotated each cell cluster ( Figure 1B) by referencing pseudotime analysis ( Figure 1C) and marker gene expressions ( Figure 1D) to maintain annotation integrity with the original report. ^7^ The completely same annotation with the original report ^7^ was difficult since the original report ^7^ contained data other than days 59, 74 and 78. For example, we consider that the “Late RPC” cluster in the original report ^7^ roughly belonged to the RPC1–5 clusters in this study while the “Early RPC” both in the original report ^7^ and this study corresponded to naïve cells with expressions of *VIM* (vimentin) and some RPC-markers such as *SFRP2* and *CCND1.* ^7^ Concretely, RPCs, early RPCs, RGCs, amacrine cells, PR/BC precursors, horizontal cells, rod photoreceptors (ROD), cone photoreceptors (CONE) and Muller glia were identified in the dataset. We then screened *NOTCH1*, *NOTCH2*, *NOTCH3* and *NOTCH4* mRNA expressions in each cell cluster and found that *NOTCH1*, *NOTCH2* and *NOTCH3* were expressed in RPCs and rapidly decreased in more differentiated cells ( Figure 1D, highlighted by the red box) ( Figure 1E). This result was consistent with the literature. ^12^ ^–^ ^14^ Since *NOTCH2* has been suggested for pigmental epithelium ^12^ ^,^ ^13^ and ciliary body ^15^ except for its expression in PRCs at a slightly detectable level, ^14^ we determined to focus on *NOTCH1* and *NOTCH3* in the following analysis. To examine the epigenetic change in the rapid decrease of *NOTCH1* and *NOTCH3* mRNA expressions, we proceeded to single-cell ATAC-seq re-analysis of the same dataset. ^7^ The cell labels were transferred from the single-cell RNA-seq data using the “FindTransferAnchors” function in the Signac pipeline. The UMAP landscape of sampled cells ( Figure 2) and the cell numbers for each cluster (“Additional_Table_1.xlsx”) suggested sufficient cell numbers in each differentiation stage for detailed analysis. We performed chromatin accessibility analysis along with co-accessible peak analysis in the *NOTCH1* and *NOTCH3* loci to screen *cis*-regulatory elements (CREs) ( Figure 3A and 3B). We observed chromatin remodeling in the *NOTCH1* locus especially in chr9: 136550000–136600000 and less prominent chromatin remodeling in the *NOTCH3* locus in chr19: 15100000–15150000, and identified multiple open chromatin regions in the *NOTCH1* and *NOTCH3* loci that were corresponded to those mRNA expressions (“Additional_Table_2.csv” for the individual peak region in the *NOTCH1* locus and their z-scores, “Additional_Table_4.csv” for the individual peak region in the *NOTCH3* locus and their z-scores). We also investigated the *NOTCH2* and *NOTCH4* loci and obtained multiple open chromatin regions even in the *NOTCH4* locus despite its low mRNA expression, raising the need for additional examination in interpreting co-accessible peak analysis (“Additional_Figure_1.tif” for the coverage plots, “Additional_Table_3.csv” for the individual peak region in the *NOTCH2* locus and their z-scores, and “Additional_Table_5.csv” for the individual peak region in the *NOTCH4* locus and their z-scores). To obtain functionally relevant TFs for further analysis on chromatin remodeling, we conducted mRNA expression analysis ( Figure 1D, highlighted by the red box, and 1F) and JASPAR motif enrichment analysis (“Additional_Table_6.csv”) for TFs known to act in RPCs (LHX2, ^1^ PAX6, ^2^ RAX ^3^ and VSX2 ^4^) (the JASPAR motifs are available in “Additional_Figure_2.tif”). JASPAR motif enrichment analysis confirmed the TFBS enrichment on a genome-wide basis for these TFs in RPCs, supporting the validity of regarding these TFs as functional TFs in RPCs. Indeed, these TFs were expressed in the RPCs ( Figure 1F) although *PAX6* expression was also observed in more differentiated cells. We then searched for their TFBSs specifically in the *NOTCH1* and *NOTCH3* loci using the FIMO tool ^45^ in the MEME Suite. ^46^ We found multiple TFBSs of PAX6 and VSX2 in the *NOTCH1* and *NOTCH3* loci (“Additional_Table_7.csv” for the *NOTCH1* locus and PAX6, “Additional_Table_8.csv” for the *NOTCH1* locus and VSX2, “Additional_Table_9.csv” for the *NOTCH3* locus and PAX6, “Additional_Table_10.csv” for the *NOTCH3* locus and VSX2). Among these TFBSs, we found one PAX6 TFBS in the *NOTCH1* and *NOTCH3* loci that were partially overlapped with the open chromatin regions identified in the co-accessible peak analysis (Chr9: 136494138–136494151 in the *NOTCH1* locus, and Chr19: 15185793–15185806 in the *NOTCH3* locus). Unfortunately, we did not find any TFBSs for VSX2 in the candidate open chromatin regions neither in the *NOTCH1* and *NOTCH3* loci. Although it was technically difficult to specifically examine the binding of PAX6 to the identified chromatin regions (Chr9: 136494138–136494151 in the *NOTCH1* locus, and Chr19: 15185793–15185806 in the *NOTCH3* locus), we made our best to examine the TF-TFBS binding status by footprinting analysis on a genome-wide basis. Footprinting analysis suggested active TF-TFBS binding for LHX2, RAX and VSX2 in RPCs ( Figure 4A, Figure 4C, Figure 4D). This analysis suggested that PAX6 primarily bound to its TFBS primarily in RGCs (RGC1, RGC2, RGC3 and RGC4) ( Figure 4B) although this binding might also occur in RPC4 that marked a later stage of RPC differentiation judged by the UMAP analysis ( Figure 1B) and pseudotime analysis ( Figure 1C) of our single-cell RNA-seq. Given that Notch1 maintains pluripotency, ^20^ ^–^ ^22^ attenuation of Notch1 signaling, which might be influenced by loss of PAX6 binding to its TFBS in the *NOTCH1* locus due to chromatin remodeling at later stages during RPC differentiation, could be of some biological importance. Taken together, we consider that footprinting analysis supports, at least does not jeopardize, the notion that PAX6 binds to their accessible TFBSs in the *NOTCH1* and *NOTCH3* loci although additional experiments such as deletion experiments as in Thomas et al., (2022) ^7^ or CUT&Tag for PAX6 are required to confirm this notion. In summary, we suggested that chromatin accessibility in the *NOTCH1* and *NOTCH3* loci changes during RPC differentiation and identified candidate TF-TFBS combinations for PAX6 possibly associated with these changes.

**Figure 1.**
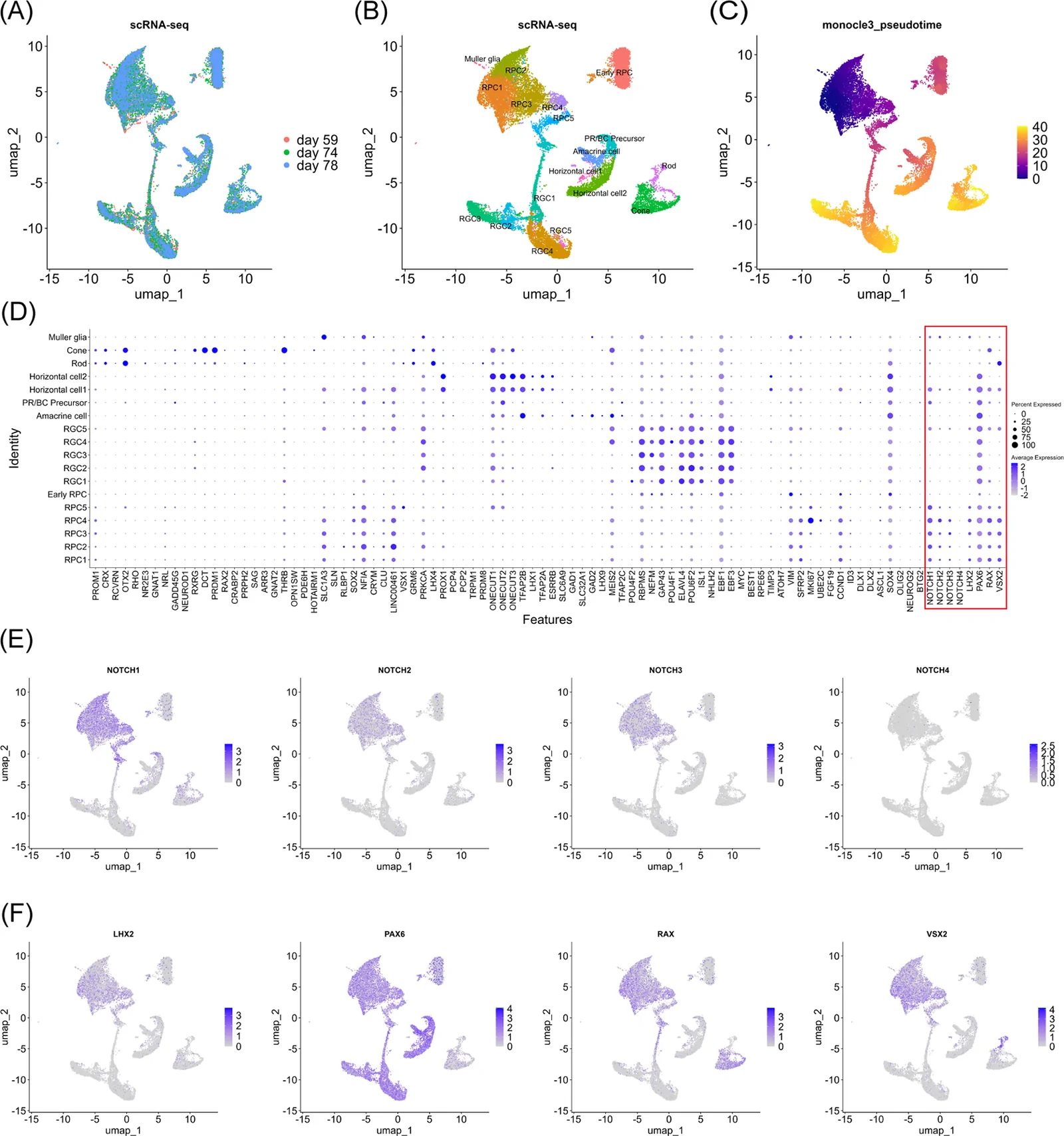
Notch and associated gene expressions during RPC differentiation. (A) UMAP analysis of the single-cell RNA-seq data of the samples at day 59, 74 and 78. (B) UMAP analysis of the single-cell RNA-seq data identified 18 clusters. Note that RPC stands for retinal progenitor cell, RGC stands for retinal ganglion cell, and PR/BC Precursor stands for photoreceptor and bipolar cell progenitor cells. (C) Monocle3 pseudotime analysis for clarifying the differentiation status. (D) Dot plot of marker gene expressions along with Notch genes and RPC-associated TFs ( *LHX2*, *PAX6*, *RAX* and *VSX2*) that were highlighted by the red box. (E) Feature plot of Notch gene expressions *.* (F) Feature plot of RPC-associated TF expressions.

**Figure 2.**
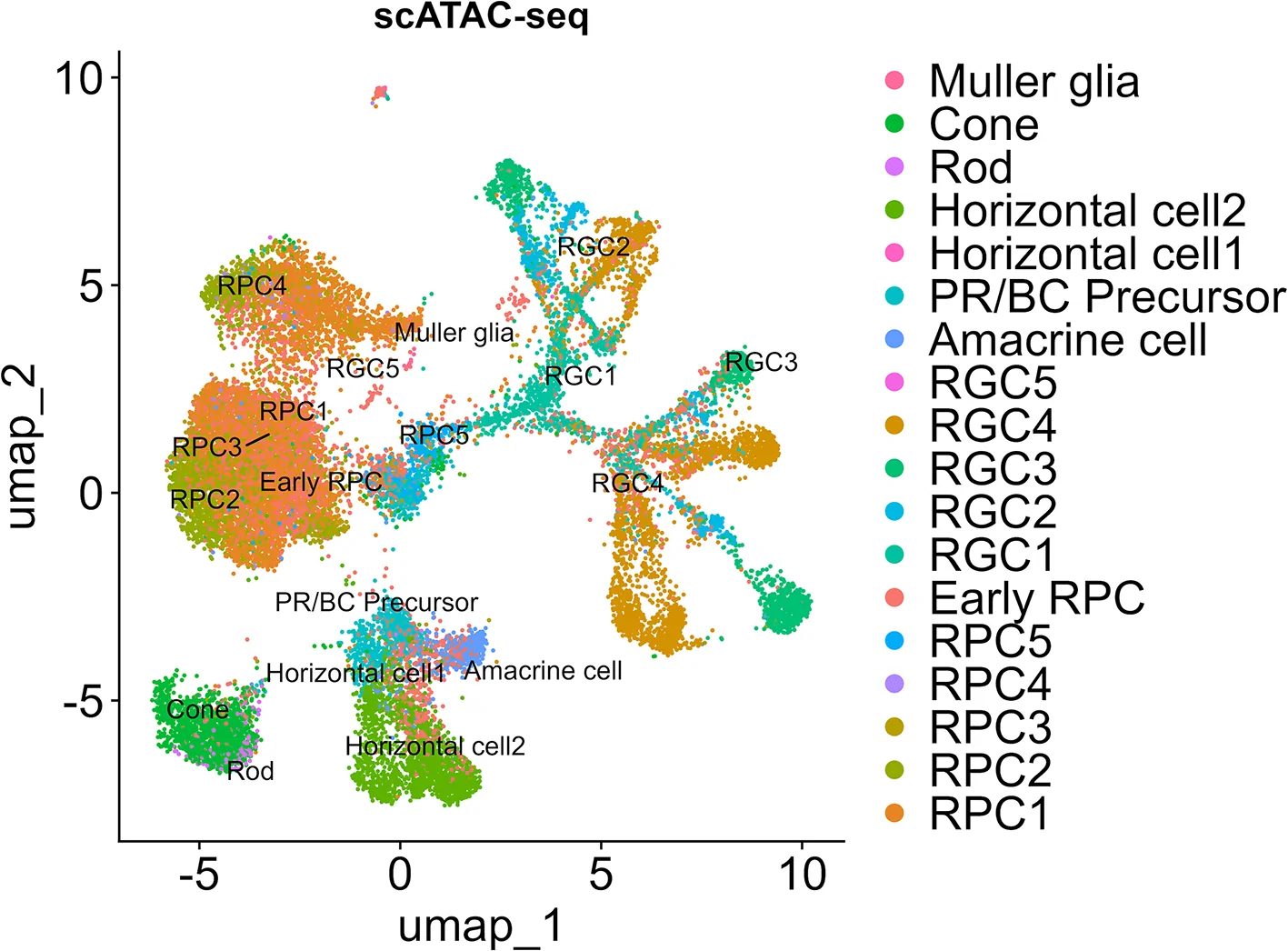
UMAP analysis of single-cell ATAC-seq data. The cell labels for single-cell ATAC-seq data are consistent with the single-cell RNA-seq data.

**Figure 3.**
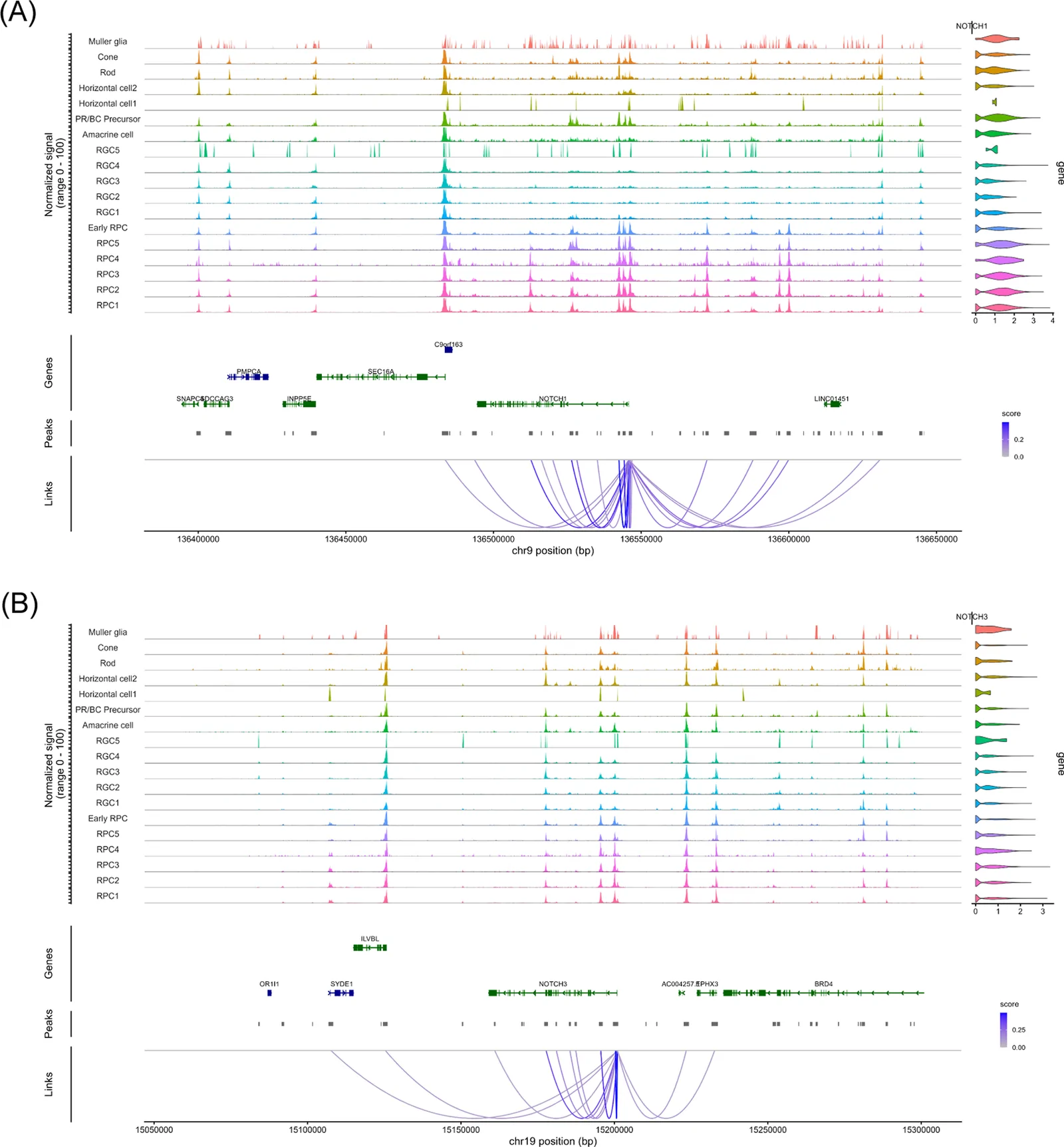
Co-accessible peak analysis of the *NOTCH1* and *NOTCH3* loci. (A) The coverage plot of the 100 kb upstream and downstream chromatin regions of *NOTCH1.* The upstream of the gene is on the right. The peaks linked to *NOTCH1* expression are indicated by strings. (B) The coverage plot of the 100 kb upstream and downstream chromatin regions of *NOTCH3.* The upstream of the gene is on the right. The peaks linked to *NOTCH3* expression are indicated by strings.

**Figure 4.**
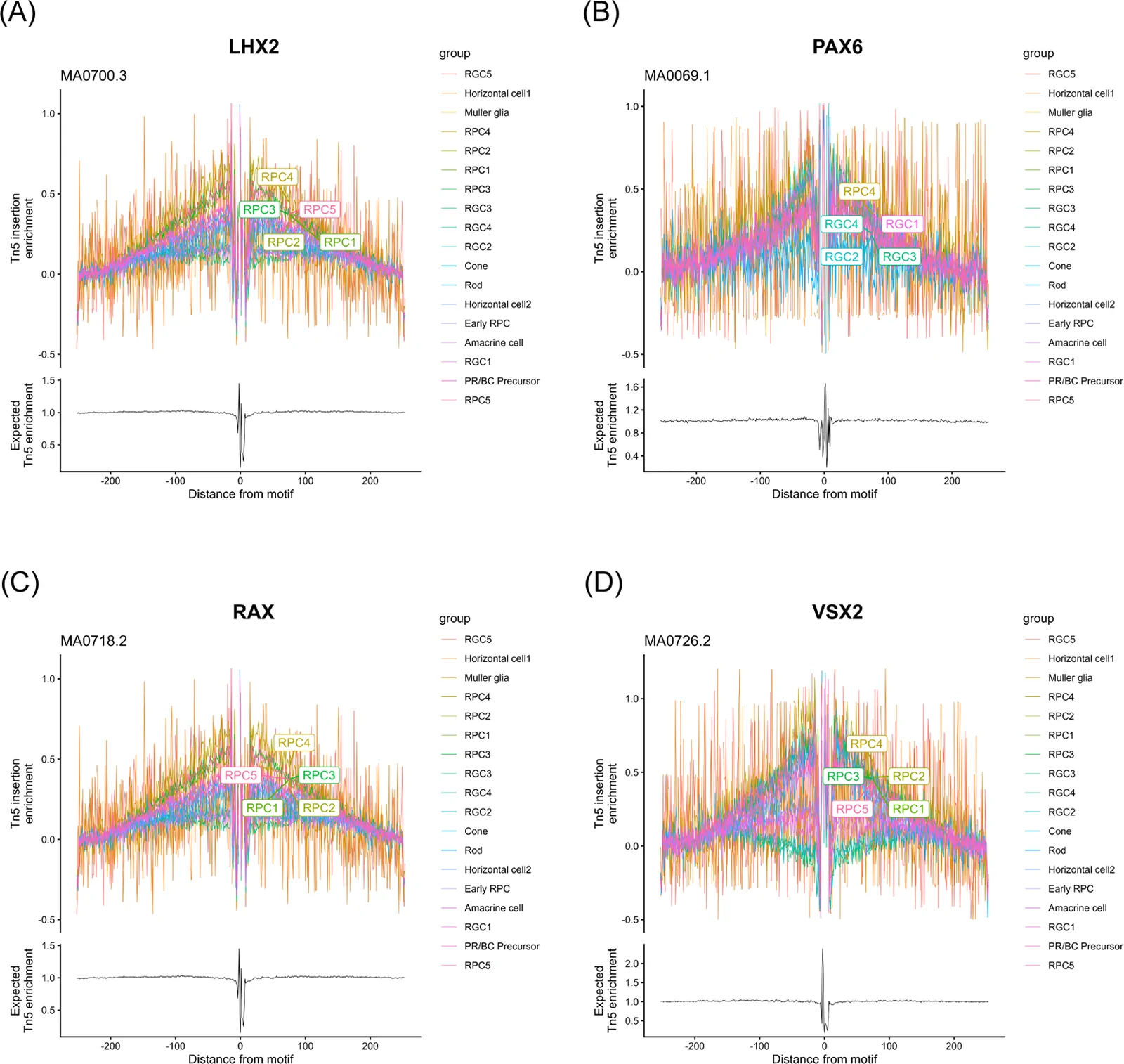
Footprinting analysis for RPC-associated TFs. Footprinting analysis with labeling of the top five clusters. (A) LHX2 motif (JASPAR matrix ID: MA0700.3). (B) PAX6 motif (JASPAR matrix ID: MA0069.1). (C) RAX motif (JASPAR matrix ID: MA0718.2). (D) VSX2 motif (JASPAR matrix ID: MA0726.2).

## Discussion

The involvement of Notch signaling in cell fate choices is well documented, including in *Drosophila* neurogenesis ^48^ and mammalian biliary development. ^49^ Although the regulation of Notch receptor expression is necessary for these processes, to the best of our knowledge, few studies have used genome-wide investigations of the underlying molecular mechanisms. To examine chromatin remodeling in such regulatory mechanisms, we re-analyzed a single-cell RNA-seq and ATAC-seq dataset from developing retinas in which differentiation trajectories were well characterized. By re-analyzing data from three human fetal retinal samples, we observed chromatin remodeling in the *NOTCH1* locus and less prominently in the *NOTCH3* locus ( Figure 3). The analyzed genomic region (100 kb upstream and downstream of each gene) contained multiple binding motifs for RPC-associated transcription factors (PAX6 and VSX2) predicted by FIMO tool ^45^ By comparing chromatin accessible regions and identified TFBSs, we found some common genomic regions in the *NOTCH1* and *NOTCH3* loci for PAX6 (Chr9: 136494138-136494151 in the *NOTCH1* locus, and Chr19: 15185793-15185806 in the *NOTCH3* locus). Importantly, the identified region in the *NOTCH1* locus (Chr9: 136494138-136494151) also showed relatively high chromatin accessibility in the “Late Progenitors” cluster among the others in the UCSC browser tracks ( http://genome.ucsc.edu/s/CherryLab/Nuclear_EyeBrowser_TrackHub ) that has been prepared by Thomas et al. ^7^ Although the original report of this multi-omics dataset ^7^ provides comprehensive data on candidate CREs including those for *NOTCH2NLR* and *NOTCH2NLC*, candidate CREs for *NOTCH1* and *NOTCH3* were not reported. Even though the PAX6 TFBSs identified in this study require additional loss-of-function analysis to confirm their functionalities, this finding strengthens the biological meaning of chromatin remodeling in the *NOTCH1* and *NOTCH3* loci during RPC differentiation. Given that PAX6 was expressed even after exit from RPCs ( Figure 1F) and actively bound to its TFBSs in some RGC populations as judged from footprinting analysis ( Figure 4B), chromatin remodeling might disrupt PAX6 binding on the *NOTCH1* and *NOTCH3* loci. It is, however, noteworthy that chromatin remodeling at the above-mentioned genomic regions (Chr9: 136494138-136494151 in the *NOTCH1* locus, and Chr19: 15185793-15185806 in the *NOTCH3* locus) might reflect global epigenetic changes at exit from RPCs to more differentiated cells since JASPAR motif enrichment analysis also showed enrichment of other TFs (LHX2, RAX and VSX2) in the RPCs. Despite these limitations of observation study, we believe that this study provides biologically important idea on the epigenetic regulation of *NOTCH1* and *NOTCH3* expressions during normal development of the eye.

Identification of *PAX6*’s TFBS in the *NOTCH1* locus that was accessible in RPCs might have some links with clinical cases as well since haploinsufficiency of this gene has been linked to aniridia, ^50^ an ophthalmological disease often accompanied by fovea hypoplasia. AAV-mediated *PAX6* overexpression in the *Small eye* ( *Sey*) mouse ^51^ ^,^ ^52^ (a mouse model of aniridia) rescued thickness of the ganglion cell layer, which was preceded by increased *Notch1* expression ^53^ although the timing of this overexpression (postnatal day 21) mimicked the average age of diagnosis in human patients (22.1 months) ^54^ and was much later than the timing of the fetal samples re-analyzed in this study. Nevertheless, given postnatal maturity of the eye ^55^ and *Notch1* expression in proliferating ( *MKI67*-expressing) RPCs ^3^ (also RPC4 in Figure 1D in this study), identification of the *PAX6* TFBS subject to chromatin remodeling in the *NOTCH1* locus would have some clinical importance despite this mismatch in analyzed timings. Concretely, since *PAX6*’s DNA binding affinity is so delicate that disease-causing *PAX6* mutations alter rather than ablate this protein-DNA binding ^56^ or need additional factors other than alterations in their DNA-binding affinities for their roles in pathogenesis, ^57^ our identification of *PAX6* TFBS in the *NOTCH1* locus as well as that in the *NOTCH3* locus would provide research cues for clarifying biochemical basis of variabilities in mutated *PAX6*’s DNA binding affinities from the DNA sequence perspective instead of the protein perspective.

We declare another technical limitation in single-cell ATAC-seq re-analysis. We observed an unexpectedly small number of peaks (“Additional_Figure_1.tif”) in the *NOTCH2* locus. We cannot explain the discrepancy between *NOTCH2* mRNA expression and the barely detectable peaks although the distal regions (such as chr1: 120739293–120739409 and chr1: 120737666–120737782) identified in Thomas et al., (2022) ^7^ might account for mRNA expression. Nevertheless, this result itself has little association with other Notch loci, and thus, does not reduce the validity of the analysis into *NOTCH1* and *NOTCH3.* It is notable that a small amount of *NOTCH2* mRNA may be stabilized by a post-transcriptional mechanism via a small non-coding regulatory RNA (CAT1), which reportedly promotes stabilization of *Notch2* mRNA, although this is beyond the scope of this study. ^58^

### Ethical considerations

This study does not generate new data from human. Therefore, we consider that there are no special requirements on recruitment and publication. In addition, this study does not involve analysis of animals and plants.

### Consent for publication

Not applicable.

## Authors’ contributions

Y. W.: Data Curation, Formal Analysis, Software, Visualization, Writing – Original Draft Preparation.

S.K.: Writing – Original Draft Preparation.

T.N.: Writing – Original Draft Preparation.

S.T.: Supervision, Writing – Review & Editing.

M.Y.: Conceptualization, Formal Analysis, Funding Acquisition, Methodology, Project Administration, Software, Writing – Original Draft Preparation.

## Data Availability

A single-cell multiomics dataset (GSE183684) ^7^ was downloaded from the Gene Expression Omnibus database ( https://www.ncbi.nlm.nih.gov/geo/query/acc.cgi?acc=GSE183684). The original data in the present study including additional data available from: https://github.com/Yoshitokky/Notch1_eye_multiomics_Additional_File/tree/main. These original data available from: https://doi.org/10.5281/zenodo.19307715. ^59^ License: OSI approved open license software is under the terms of the Creative Commons Zero “No rights reserved” data waiver (CC0 1.0 Public domain dedication). The project contains the following underlying data:
•
Additional_Figure_1.tif. •
Additional_Figure_2.tiff. •
Additional_Table_1.xlsx. •
Additional_Table_2.csv. •
Additional_Table_3.csv. •
Additional_Table_4.csv. •
Additional_Table_5.csv. •
Additional_Table_6.csv. •
Additional_Table_7.csv. •
Additional_Table_8.csv. •
Additional_Table_9.csv. •
Additional_Table_10.csv.

## Software availability

Source code available from: https://github.com/Yoshitokky/Notch1_eye_multiomics_code/tree/main.

Archived software available from: https://doi.org/10.5281/zenodo.19282258. ^60^

License: OSI approved open license software is under the terms of the Creative Commons Zero “No rights reserved” data waiver (CC0 1.0 Public domain dedication).

The project contains the following underlying data:

• 260320_retina_chromatin_d59_before_merge.R. (code No.1)
• 260320_retina_chromatin_d74_before_merge.R. (code No.2)
• 260320_retina_chromatin_d78_before_merge.R. (code No.3)
• 260320_retina_chromatin_d59_d74_d78_merged_No0.R. (code No.4)
• 260320_retina_chromatin_d59_d74_d78_merged_No1.R. (code No.5)
• 260320_retina_chromatin_d59_d74_d78_merged_No2.R. (code No.6)
• 260320_retina_chromatin_d59_d74_d78_merged_No3.R. (code No.7)
• 260320_retina_chromatin_d59_d74_d78_merged_No4.R. (code No.8)
• 260320_retina_chromatin_d59_d74_d78_merged_No5.R. (code No.9)
• 260320_retina_chromatin_d59_d74_d78_merged_No6.R. (code No.10)
• 260320_retina_chromatin_d59_d74_d78_merged_No7.R. (code No.11)
• 260320_retina_chromatin_d59_d74_d78_merged_No8.R. (code No.12)
• 260320_retina_chromatin_d59_d74_d78_merged_No9.R. (code No.13)
• 260320_retina_chromatin_d59_d74_d78_merged_No10.R. (code No.14)
• 260320_retina_chromatin_d59_d74_d78_merged_No11.R. (code No.15)
• 260320_retina_chromatin_d59_d74_d78_merged_No12.R. (code No.16)
• 260320_retina_chromatin_d59_d74_d78_merged_No13.R. (code No.17)
• 260320_retina_chromatin_d59_d74_d78_merged_No14.R. (code No.18)
• 260320_retina_chromatin_d59_d74_d78_merged_No15.R. (code No.19)
