# Expression status transition of *NOTCH1* accompanies chromatin remodeling in human early retinal progenitor cells

**DOI:** 10.12688/f1000research.159630.5

**Published:** 2026-08-21

**Authors:** Yoshitoku Watabe, Sakurako Kobayashi, Takahiro Nakayama, Satoru Takahashi, Masaharu Yoshihara

**Affiliations:** 1 College of Medicine, School of Medicine and Health Sciences, University of Tsukuba, Tsukuba, Ibaraki Prefecture, Japan; 2 Department of Anatomy and Embryology, Institute of Medicine, University of Tsukuba, Tsukuba, Japan; 3 Transborder Medical Center, Institute of Medicine, University of Tsukuba, Tsukuba, Japan; 4 Department of Primary Care and Medical Education, Institute of Medicine, University of Tsukuba, Tsukuba, Japan

**Keywords:** differentiation, epigenetics, eye development, single-cell ATAC-seq, single-cell RNA-seq

## Abstract

**Background:**

Notch signaling, especially via the Notch1 receptor, is involved in normal eye development by maintaining retinal progenitor cell state before their differentiation, although the regulatory mechanism of fine-tuned Notch1 expression is unclear.

**Methods:**

We re-analyzed publicly available human fetal retina single-cell RNA/ATAC-seq data at days 59, 74, and 78, using Seurat/Signac pipelines.

**Results:**

We observed *NOTCH1* expression in retinal progenitor cells, which diminished at later stages of differentiation. Integration of single-cell RNA-seq and ATAC-seq revealed a decrease in chromatin accessibility at the *NOTCH1* locus, accompanied by a decrease in its mRNA expression during differentiation into bipolar cells, horizontal cells, or retinal ganglion cells. Importantly, those chromatin remodeling regions contained the binding motifs of the *RAX* transcription factor, which is essential for retinal progenitor cells, Muller glia, and cones. This result suggested that the regulation of *NOTCH1* expression might be achieved by chromatin remodeling at the *NOTCH1* locus, although single-cell RNA-seq analysis suggested that *RAX* expression regulation might also contribute to *NOTCH1* expression.

**Conclusions:**

By analyzing retinal cell differentiation, this study identified a putative *RAX* binding site in the *NOTCH1* locus and raised the possibility that *NOTCH1* expression could be regulated by chromatin remodeling.

## Introduction

Signal transduction via the Notch signaling pathway depends on the expression of receptors regulated at multiple levels. In addition to intracellular machineries such as glycosylation, ^1^ ubiquitination, ^2^ and phosphorylation, ^3^ these regulations include chromatin remodeling and DNA-binding proteins, such as transcription factors and transcriptional repressors, which could regulate the levels of Notch ligands and receptors. Since fine-tuned signal transduction is necessary for development, it is important to clarify the regulatory mechanisms of receptor expression for understanding the regulation of cell differentiation and subsequent tissue development.

The retina is a good model for investigating cellular differentiation since cell fate determination is well documented. The developed retina is composed of multiple cell types, including retinal ganglion cells, amacrine cells, bipolar cells, horizontal cells, rods, cones, and glial cells (Muller glia), all of which originate from retinal progenitor cells (retinal progenitors). In addition, astrocytes and microglia exist during retinal development. Recently, a detailed molecular characterization at single-cell resolution of the developing retina was reported. ^4^ In addition, retinal progenitors are also known to express several transcription factors such as *Lhx2*, ^5^ *Pax6,* ^6^ *Rax*, ^7^ and *Vsx2.* ^8^

Notch signaling involves four Notch receptors that are expressed differently in space. In rat embryonic eyes, *Notch1* was expressed on the posterior side of the retina at embryonic days (E) 13.5 and E14.5; Notch2 was expressed in the retinal pigment epithelium throughout E12.5 and E14.5; and Notch3 was expressed throughout the retina from E12.5 to E14.5, with strong expression on the anterior side at E14.5. ^9^ ^,^ ^10^ Further analyses on *Notch2* using a knock-in mouse suggested that *Notch2* was expressed at a slightly detectable level from the E9.5 optic vesicle stage in the retinal neuroepithelium that contained retinal progenitors. ^11^ In addition, deletion of *Notch2* in outer ciliary epithelium disrupted ciliary body formation, ^12^ suggesting that this gene acts in the temporal side of the eye. Indeed, the spatial information in the mouse retina at E13.5 determined upstream regulators involved in *Hes1* induction, which acts as a hub for Notch-dependent and -independent inputs in retinal progenitors. ^13^ Notably, *Notch4* was expressed in arterial endothelial cells rather than retinal progenitors and their descendants in the retina. ^14^

Notch signaling is also involved in the complex differentiation of retinal progenitors into differentiated cells. Loss-of-function of Notch common co-actors *Rbpj* ^15^ and *Maml* ^16^ increased *Crx*-expressing photoreceptor precursor cells and cone/horizontal cell-restricted retinal progenitors, respectively. More specifically, *Notch1* has been shown to inhibit the rod fate and maintain pluripotency of retinal progenitors. ^17^ ^−^ ^19^ This notion is also supported by a single-cell ATAC-seq and disease-associated 5q14.3 enhancer knockout study. ^20^ In addition, *Notch3*, as well as *Notch1*, was shown to be expressed as early as the optic vesicle stage. ^21^ Indeed, *Notch3* knockout mice showed a reduced number of retinal ganglion cells, ^22^ although zebrafish *Notch3* was also essential for Muller glial cells. ^23^ These results suggest that *Notch1* and *Notch3* act in retinal progenitors to regulate their differentiation, although it is unclear how retinal progenitor-dominant expressions of *Notch1* and *Notch3* are regulated during retinal progenitor differentiation.

A murine ophthalmological study examined global epigenetic changes and interacting transcription factors during retinal cell differentiation. ^24^ By chromVAR analysis in the Signac pipeline and CUT&Tag analysis, this study revealed that the epigenetic landscape of cell type-specific enhancers shifted during retinal cell differentiation and identified several transcription factors (e.g., *Otx2*, *Atoh7*, *Pou4f2*, and *Isl1*) that potentially work in each differentiation stage. ^24^ ChromVAR analysis identified five *Atoh7*-binding motifs in the *Notch1* locus that were specific to naïve retinal progenitors, although CUT&Tag analysis did not result in positive findings for any of these motifs. In contrast, this report also identified chromatin remodeling at retinal progenitor-specific enhancers for key transcription factors, including *Vsx2, * and showed binding of transcription factors and their binding sites for key transcription factors, including *Rax*, *Lhx2*, and *Pax6*, in naïve and transitional retinal progenitors, although specific genomic regions for these transcription factors are still lacking, as chromVAR was used. Another study also reported chromatin remodeling in the *NOTCH1* locus in humans, ^20^ although a comprehensive analysis focusing on possible epigenetic regulation of the four Notch receptor gene expressions in humans, including the *cis*-regulatory elements, is still lacking, as this study focused on retinal cell differentiation instead of Notch signaling.

The Notch loci are subject to epigenetic regulation under both normal ^25^ ^–^ ^30^ and pathological ^31^ ^–^ ^39^ conditions in a context-dependent manner. Although a single-cell RNA/ATAC-seq dataset from developing retinas, in which differentiation trajectories were well characterized, is available, and the accessibility of the *NOTCH1* locus is known to decrease over differentiation, ^20^ how this chromatin remodeling affects *NOTCH1* expression is still unknown. To gain insight into chromatin remodeling and possible *cis*-regulatory elements responsible for regulation of the Notch receptor gene expression, we re-analyzed a publicly available multi-omics dataset of single-cell RNA-seq and single-cell ATAC-seq of human fetal retinas. ^20^

## Methods

 To perform fine-grained annotation of the single-cell RNA-seq and ATAC-seq data, we downloaded an h5ad file, ^4^ which includes cell annotations from detailed molecular characteristics analysis of embryonic retinal cells from the CZ CELLxGENE Discover platform ( https://cellxgene.cziscience.com/collections/c11009b8-b113-4a99-9890-78b2f9df9d79). The h5ad file was converted into an RDS file and then analyzed in the Seurat pipeline. ^40^

A single-cell RNA/ATAC-seq dataset (GSE183684) ^20^ was downloaded from the Gene Expression Omnibus ( https://www.ncbi.nlm.nih.gov/geo/). Data from the earliest three days (day 59, 74, and 78) in this dataset were used to balance computational burden and the number of retinal progenitors. The data were processed in the pipelines using Seurat and Signac. ^41^ Detailed session information for each step is provided in the archived code (See Data availability section). Integration of the single-cell RNA-seq data and the single-cell ATAC-seq data was conducted using the “FindTransferAnchors” function of Signac. We used the FIMO tool ^42^ in the MEME Suite, ^43^ referencing the JASPAR motif database ( https://jaspar.elixir.no/) and human genome assembly hg38 in the University of California, Santa Cruz genome browser ( https://genome.ucsc.edu/) to search transcription factor binding motifs.

## Results

We started by analyzing the single-cell RNA-seq data ^4^ that we will use for annotation in the other single-cell RNA/ATAC-seq data. ^20^ Uniform manifold approximation and projection (UMAP) analysis showed complexly ramified cell clusters (  Figure 1A), and in concordance with the original report, ^4^ we identified *SOX2* and *MKI67*-double-positive Retinal progenitors (  Figure 1B-C). The cell number in each cluster is available in Additional Table 1. We performed dot plot analysis of the marker genes ( *SOX2* and *MKI67* for Retinal progenitors; *RLBP1* for Muller glia; *PAX6* for Retinal ganglion cell/Amacrine cell/Horizontal cell fate; *RCVRN*, *OTX2*, *PDE6H*, *NRL*, and *RHO* for Rods and Cones; *VSX2* for Bipolar cells; *TFAP2A*, *TFAP2B* for Amacrine cells; *ONECUT2* for Horizontal cells; *POU4F2* and *RBPMS* for Retinal ganglion cell) according to the original report ^4^ to provide the average expression levels and proportion of these marker-expressing cells to guarantee the validity of annotation (  Figure 1D). Importantly, some of the markers were expressed in multiple cell clusters. We then screened the four Notch receptor genes and found that *NOTCH1* was expressed in the Retinal progenitors (log2 fold change to background: 1.09, adjusted p-value: 0), Transitory (log2 fold change to background: 1.32, adjusted p-value: 0), and Muller glia (log2 fold change to background: 1.43, adjusted p-value: 0) cell clusters. The other Notch receptor genes were also expressed in the Retinal progenitors, although the expression was sparse, prompting us to focus on *NOTCH1.* We also examined the expression of *RAX*, *PAX6*, *LHX2*, and *VSX2.* *RAX* was enriched in the Retinal progenitors (log2 fold change to background: 0.62, adjusted p-value: 0), Transitory (log2 fold change to background: 0.87, adjusted p-value: 0), Muller glia (log2 fold change to background: 0.72, adjusted p-value: 1.66 x 10 ^−149^), Cone precursors (log2 fold change to background: 0.59, adjusted p-value: 7.02 x 10 ^−35^), Cones (log2 fold change to background: 1.31, adjusted p-value: 0), and Astrocytes (log2 fold change to background: 0.48, adjusted p-value: 5.36 x 10 ^−3^) cell clusters. *PAX6* was also enriched in Retinal progenitors (log2 fold change to background: 0.15, adjusted p-value: 1.84 x 10 ^−54^), Transitory (log2 fold change to background: 0.23, adjusted p-value: 2.34 x 10 ^−163^), Amacrine cells (log2 fold change to background: 1.20, adjusted p-value: 0), Horizontal cells (log2 fold change to background: 1.13, adjusted p-value: 0), Retinal ganglion cells (log2 fold change to background: 0.35, adjusted p-value: 1.15 x 10 ^−160^), and Muller glia (log2 fold change to background: 0.18, adjusted p-value: 6.83 x 10 ^−39^) cell clusters. *LHX2* was enriched in Retinal progenitors (log2 fold change to background: 0.95, adjusted p-value: 0), Transitory (log2 fold change to background: 1.43, adjusted p-value: 0), Muller glia (log2 fold change to background: 0.94, adjusted p-value: 1.59 x 10 ^−163^), Astrocytes (log2 fold change to background: 1.15, adjusted p-value: 5.20 x 10 ^−20^) cell clusters. *VSX2* was enriched in Retinal progenitors (log2 fold change to background: 0.42, adjusted p-value: 2.12 x 10 ^−175^), Transitory (log2 fold change to background: 1.07, adjusted p-value: 0), Bipolar cells (log2 fold change to background: 2.07, adjusted p-value: 0), Muller glia (log2 fold change to background: 0.60, adjusted p-value: 1.60 x 10 ^−91^) cell clusters. All the enriched gene information is available in Additional Table 2. For the following single-cell ATAC-seq analysis, we provide UMAP results highlighting Retinal progenitors (  Figure 1E) and Transitory ( Figure 1F) cell clusters, and feature plots for *NOTCH1* (  Figure 1G) and *RAX* (  Figure 1H). We note that Retinal progenitors and Transitory cell clusters showed similar distributions in the UMAP, in which *NOTCH1* and *RAX* were expressed.

**Figure 1.**
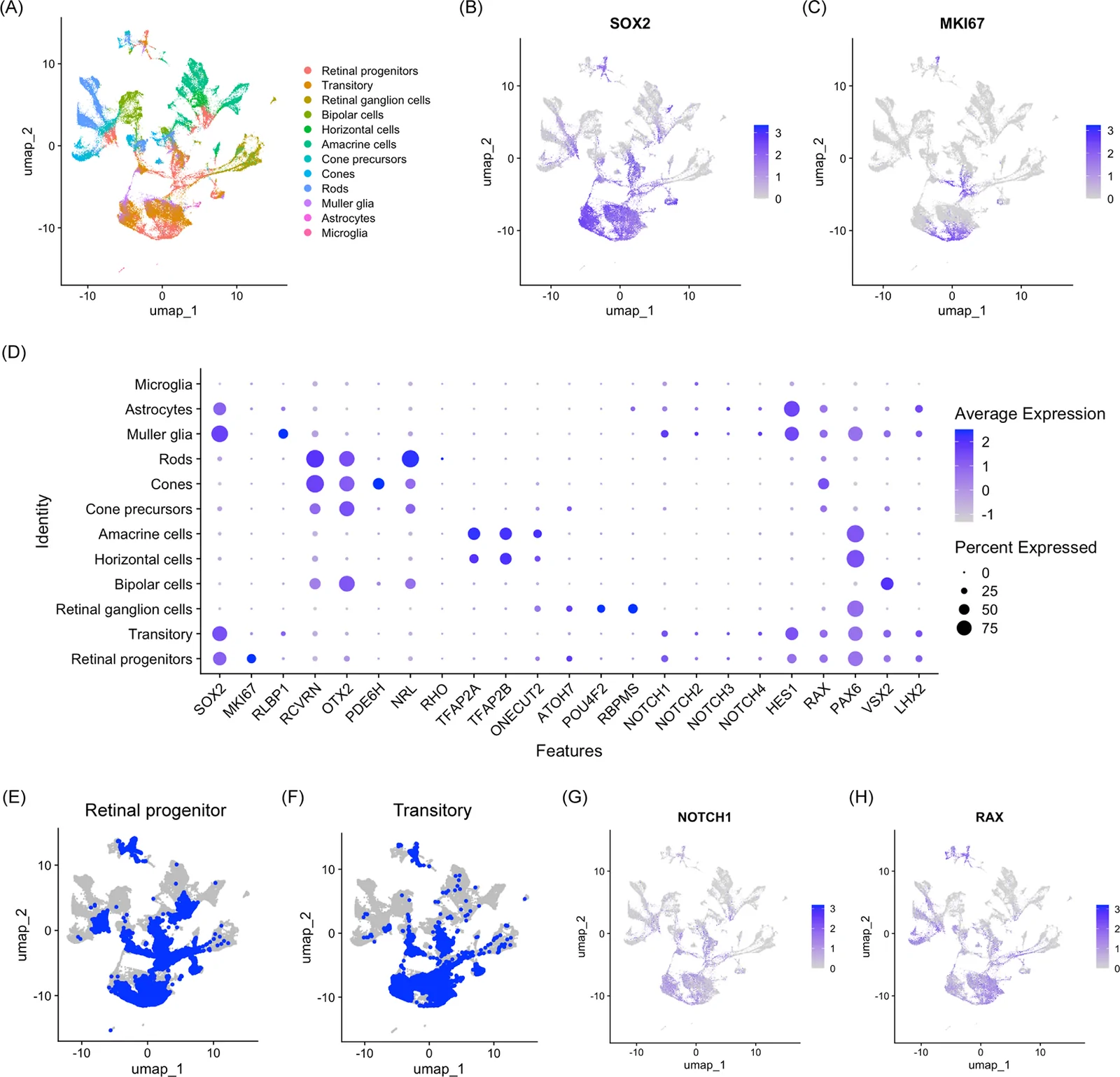
Single-cell RNA-seq analysis of a dataset for annotation. (A) UMAP analysis of the single-cell RNA-seq data ^4^ for annotation of the other single-cell RNA/ATAC-seq dataset. ^20^ (B) Feature plot for *SOX2* expression. (C) A feature plot for *MKI67* expression. (D) A dot plot of marker gene expressions along with Notch genes and Retinal progenitors-associated TFs ( *RAX*, *PAX6*, *LHX2*, and *VSX2*). (E) Clear representation of Retinal progenitors in the UMAP result. (F) Clear representation of Transitory cell clusters in the UMAP result. (G) *NOTCH1* expression in the UMAP result. (H) *RAX* expression in the UMAP result.

Next, we examined single-cell RNA-seq data from human fetal retinas at day 59, 74, and 78 from GSE183684. ^20^ The cell number in each cluster is available in Additional Table 3. After using the “RunHarmony” function ^44^ with the Seurat pipeline to reduce batch effects, we annotated the cells according to the earlier single-cell RNA-seq data ^4^ using the “FindTransferAnchor” function with the Seurat pipeline. This data also contained all types of the aforementioned cell clusters ( Figure 2A). Indeed, we observed *SOX2*/ *MKI67*-double-positive Retinal progenitors (  Figure 2B-C). We also provided a dot plot for characteristic gene expression for each cell cluster (  Figure 2D). As expected, screening of the Notch receptor genes suggested prominent *NOTCH1* expression in the Retinal progenitors (log2 fold change to background: 1.26, adjusted p-value: 0), Transitory (log2 fold change to background: 1.03, adjusted p-value: 1.88 x 10 ^−231^), and Muller glia (log2 fold change to background: 1.45, adjusted p-value: 1.50 x 10 ^−106^) cell clusters. In addition, *RAX* was enriched in Retinal progenitors (log2 fold change to background: 0.64, adjusted p-value: 1.32 x 10 ^−131^), Transitory (log2 fold change to background: 0.81, adjusted p-value: 3.88 x 10 ^−153^), Muller glia (log2 fold change to background: 0.38, adjusted p-value: 2.03 x 10 ^−10^), Cone precursors (log2 fold change to background: 0.95, adjusted p-value: 6.79 x 10 ^−82^), Cones (log2 fold change to background: 1.43, adjusted p-value: 3.67 x 10 ^−107^), and Rods (log2 fold change to background: 0.96, adjusted p-value: 8.94 x 10 ^−16^) cell clusters. *PAX6* was enriched in Horizontal cells (log2 fold change to background: 0.84, adjusted p-value: 2.53 x 10 ^−122^) and Retinal ganglion cells (log2 fold change to background: 0.21, adjusted p-value: 1.98 x 10 ^−43^) cell clusters but not in Retinal progenitors or Transitory cell clusters, possibly owing to its wide expression across the cell clusters. *LHX2* was enriched in Retinal progenitors (log2 fold change to background: 0.87, adjusted p-value: 4.90 x 10 ^−169^), Transitory (log2 fold change to background: 1.03, adjusted p-value: 1.62 x 10 ^−153^), Muller glia (log2 fold change to background: 0.21, adjusted p-value: 1.31 x 10 ^−3^), Amacrine cells (log2 fold change to background: 0.49, adjusted p-value: 8.75 x 10 ^−2^), and Astrocytes (log2 fold change to background: 1.29, adjusted p-value: 1.16 x 10 ^−4^) cell clusters. *VSX2* was enriched in Retinal progenitors (log2 fold change to background: 0.67, adjusted p-value: 3.82 x 10 ^−152^), Transitory (log2 fold change to background: 1.14, adjusted p-value: 6.82 x 10 ^−290^), Muller glia (log2 fold change to background: 0.75, adjusted p-value: 2.38 x 10 ^−44^), Amacrine cells (log2 fold change to background: 0.41, adjusted p-value: 1.50 x 10 ^−8^), Bipolar cells (log2 fold change to background: 2.64, adjusted p-value: 7.01 x 10 ^−152^) cell clusters. All the enriched gene information is available in Additional Table 4. For the following single-cell ATAC-seq analysis, we provide UMAP results highlighting Retinal progenitors (  Figure 2E) and Transitory ( Figure 2F) cell clusters, and feature plots for *NOTCH1* (  Figure 2G) and *RAX* (  Figure 2H). Similar to the previous data (  Figure 1E-H), feature plot analysis suggested that *NOTCH1* and *RAX* were expressed in Retinal progenitors and Transitory cell clusters.

**Figure 2.**
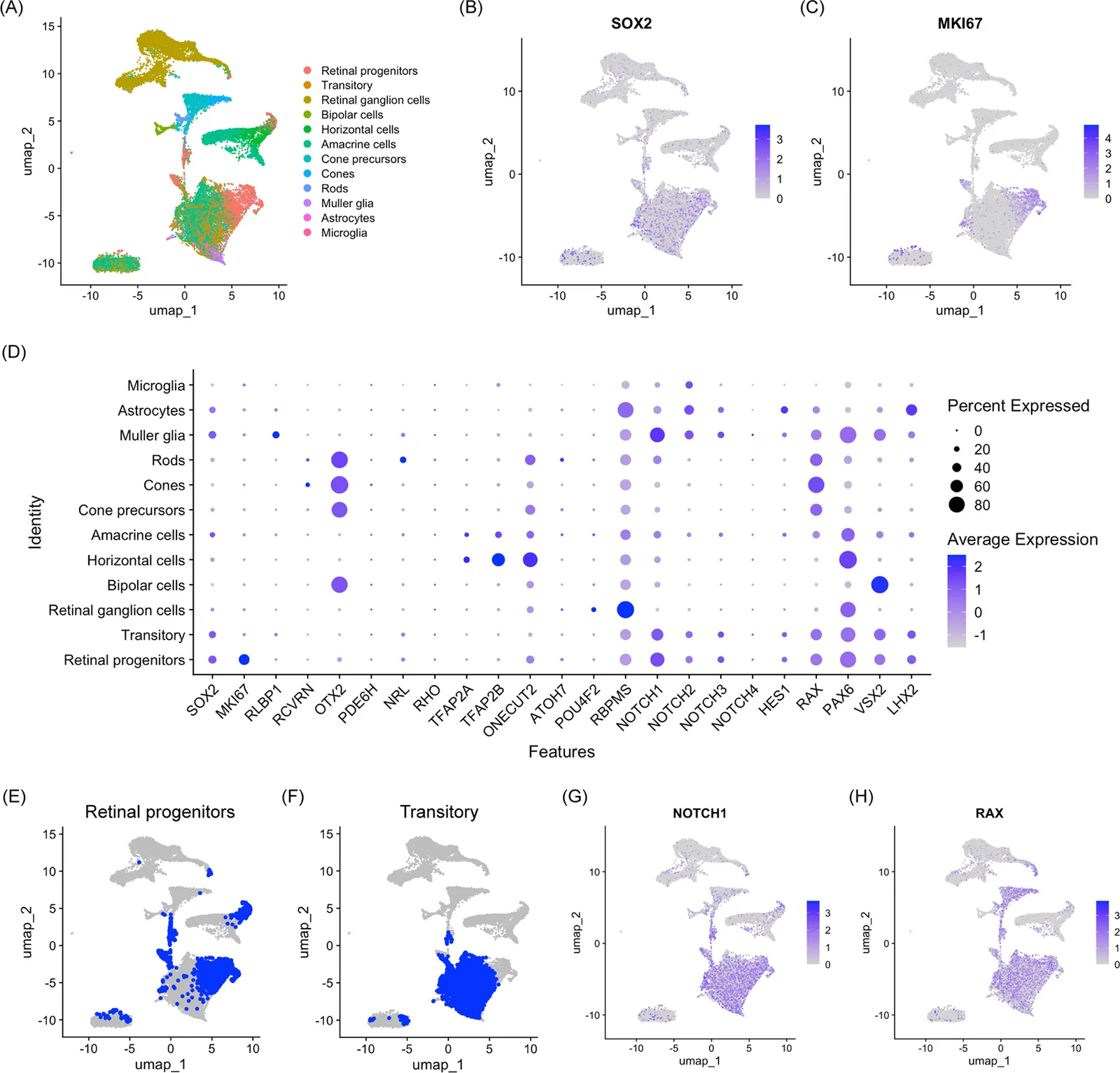
Single-cell RNA-seq analysis of human fetal retina at day 59, 74, and 78. (A) UMAP analysis of the single-cell RNA-seq data of a single-cell RNA-seq dataset. ^20^ (B) A feature plot for *SOX2* expression. (C) A feature plot for *MKI67* expression. (D) A dot plot of marker gene expression along with Notch genes and retinal progenitor-associated TFs ( *RAX*, *PAX6*, *LHX2*, and *VSX2*). (E) Clear representation of Retinal progenitors in the UMAP result. (F) Clear representation of Transitory cell clusters in the UMAP result. (G) *NOTCH1* expression in the UMAP result. (H) *RAX* expression in the UMAP result.

To examine epigenetic changes underlying *NOTCH1* expression dynamics, we examined single-cell ATAC-seq data from human fetal eyes at day 59, 74, and 78 from GSE183684. ^20^ UMAP analysis confirmed that this data contains Retinal progenitors and Transitory cell clusters, along with other differentiated cell clusters, including Cones (  Figure 3A-C). The cell number in each cluster is available in Additional Table 5. We examined Tn5 insertion frequency in the genomic *NOTCH1* region, including 100 kb upstream and downstream, with the Signac pipeline ( Figure 3D). To identify the statistically significant Tn5 insertion sites, we used the “Links” function in the Signac pipeline to obtain z-scores and p-values (Additional Table 6). To examine the transcription factor binding sites of *RAX*, *PAX6*, *LHX2*, and *VSX2* in those Tn5 insertion sites, we used the FIMO tool and identified potential transcription factor binding sites (Additional Table 7). By comparing the Tn5 insertion sites and the potential transcription factor binding sites, we found a statistically significant Tn5 insertion site in the genomic *NOTCH1* region (chr9: 136542126–136542839) containing a transcription factor binding site (chr9: 136542636–136542645) for *RAX*, but not for *PAX6*, *LHX2*, and *VSX2.* This region is indicated with an arrowhead in the whole genomic *NOTCH1* region ( Figure 3D) and the focused region (  Figure 3E). We also performed footprinting analysis and found that RAX-binding is frequently observed in Retinal progenitors, Transitory, and Muller glia cell clusters (  Figure 3F), although this analysis is genome-wide and not specific to the genomic *NOTCH1* region. Importantly, the Tn5 insertion event is frequent in Retinal progenitors, Transitory, Amacrine cells, Cone precursors, Cones, Rods, and Muller glia compared with Bipolar cells, Horizontal cells, and Retinal ganglion cells. As *RAX* is not a classic pioneer factor, in contrast to *SOX2* and *PAX6*, this observation raises the possibility that fine-tuned *RAX* and *NOTCH1* action in Retinal progenitors, Transitory, Amacrine cells, Cone precursors, Cones, Rods, and Muller glia is supported by chromatin remodeling, along with regulated *RAX* expression.

**Figure 3.**
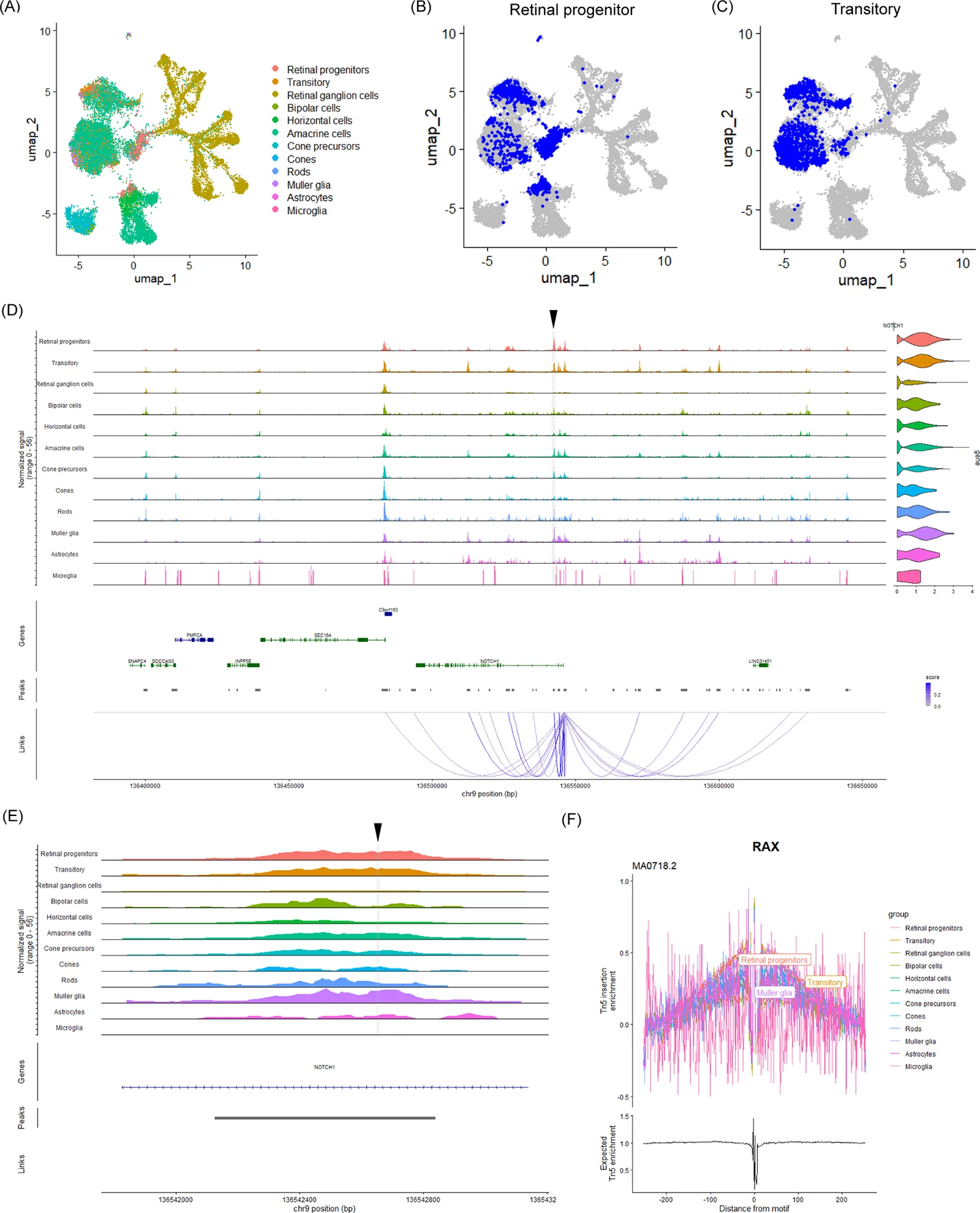
Single-cell ATAC-seq analysis of human fetal retina at day 59, 74, and 78. (A) UMAP analysis of the single-cell ATAC-seq data. ^20^ (B) Clear representation of Retinal progenitors in the UMAP result *.* (C) Clear representation of Transitory cell cluster in the UMAP result. (D) A peak plot for the *NOTCH1* locus with the 100 kb upstream and downstream. The arrowhead shows the chromatin remodeling region in the *NOTCH1* locus, which contains a RAX-binding site and is highlighted in (E). (E) A peak plot for the *NOTCH1* locus focusing on the chromatin remodeling region containing a *RAX*-binding site (arrowhead). (F) Footprint analysis revealed frequent RAX binding in Retinal progenitors, Transitory, and Muller glia.

## Discussion

The involvement of Notch signaling in cell fate choices is well documented, including in *Drosophila* neurogenesis ^45^ and mammalian biliary development. ^46^ A computer simulation study suggested that the regulation of Notch receptor expression is necessary for proper signal transduction in the developing tissue. ^47^ To the best of our knowledge, few studies have used genome-wide investigations for regulatory mechanisms of Notch receptor gene expression. Although the accessibility of the *NOTCH1* locus is known to decrease during retinal cell differentiation, ^20^ how this chromatin remodeling affects *NOTCH1* expression, along with candidate *cis*-regulatory elements, is still unknown. In this study, we hypothesized that chromatin remodeling and transcription factor levels are involved and sought possible examples using a public single-cell RNA/ATAC-seq dataset. ^20^ We used an already processed dataset from another study that intensively examined molecular characteristics of developing retinal cells ^4^ to enable reliable annotation of the cells in the single-cell RNA/ATAC-seq dataset. ^20^ We observed prominent *NOTCH1* expression both in the reference and query single-cell RNA-seq datasets, although the involvement of other Notch receptors in the developing retina was also reported. ^9^ ^–^ ^14^ Therefore, in the single-cell ATAC-seq analysis, we focused on the *NOTCH1* locus, including 100 kb upstream and downstream of its coding region. We observed distinct Tn5 insertion frequencies depending on differentiation status and, importantly, identified one transcription factor binding site for *RAX* among four transcription factors implicated in retinal progenitors ( *RAX*, *PAX6, LHX2*, and *VSX2*). This RAX-binding site is open in Retinal progenitors, Transitory, Amacrine cells, Cone precursors, Cones, Rods, and Muller glia compared with Bipolar cells, Horizontal cells, and Retinal ganglion cells, which is in line with previous studies. ^7^ ^,^ ^48^

One of the transcription factors linked to *NOTCH1* is *PAX6.* *PAX6* haploinsufficiency has been linked to aniridia, ^49^ an ophthalmological disease often accompanied by fovea hypoplasia. AAV-mediated *PAX6* overexpression in the *Small eye* ( *Sey*) mouse (a mouse model of aniridia) ^50^ ^,^ ^51^ rescued ganglion cell layer thickness, which was preceded by increased *Notch1* expression. ^52^ However, the timing of this overexpression (postnatal day 21) mimicked the average age of diagnosis in human patients (22.1 months) ^53^ and was much later than the timing of the fetal samples re-analyzed in this study. It is known that *PAX6*’s DNA-binding affinity is so delicate that disease-causing *PAX6* mutations alter rather than ablate this protein-DNA binding ^54^ or require additional factors other than alterations in their DNA-binding affinities for their roles in pathogenesis. ^55^ We observed *PAX6* expression in a wide variety of cell clusters, including Retinal progenitors and Transitory cell clusters, both in the reference and query single-cell RNA-seq datasets. In embryonic stem cells, *SOX2* (one of the Retinal progenitors markers) ^4^ binds to “hidden” chromatin regions in the nucleosome, and then *PAX6* binds to the *SOX2*-bound “unwrapped” genomic regions to facilitate neural differentiation. ^56^ Expression of these pioneer factors ^57^ was indeed observed in the Retinal progenitors and Transitory cell clusters in the reference and query single-cell RNA-seq datasets. Given that *rax* upregulated *notch1* transcription, ^7^ it is possible that *PAX6* and *SOX2* unwrap multiple genomic regions before RAX binding to the *NOTCH1* locus; however, we were not able to identify *PAX6*-binding sites in the *NOTCH1* locus, although the delicate nature of *PAX6* binding might be involved. Although this point needs further investigation, it does not jeopardize the finding that chromatin remodeling occurs at the *RAX* binding site in the *NOTCH1* locus. As *RAX* expression is observed in Retinal progenitors, Transitory, Muller glia, Cone precursors, and Cones cell clusters in the two single-cell RNA-seq datasets, it is likely that *NOTCH1* expression in Retinal progenitors and Transitory cell clusters is in part regulated at the *RAX* protein level. However, it is also possible that the chromatin remodeling that we observed in this study could help fine-tune regulation of *NOTCH1* expression. In conclusion, we provide a chromatin remodeling region in the *NOTCH1* locus that contains a *RAX* binding site, raising the possibility of regulation of *NOTCH1* expression at the transcription factor protein and chromatin levels.

### Ethical considerations

This study does not generate new data from humans. Therefore, we consider that there are no special requirements on recruitment and publication. In addition, this study does not involve analysis of animals and plants.

#### Consent for publication

Not applicable.

## Authors’ contributions

Y. W.: Data Curation, Formal Analysis, Software, Visualization, Writing – Original Draft Preparation.

S.K.: Writing – Original Draft Preparation.

T.N.: Writing – Original Draft Preparation.

S.T.: Supervision, Writing – Review & Editing.

M.Y.: Conceptualization, Formal Analysis, Funding Acquisition, Methodology, Project Administration, Software, Writing – Original Draft Preparation.

## Data Availability

A single-cell multiomics dataset (GSE183684) ^20^ was downloaded from the Gene Expression Omnibus database ( https://www.ncbi.nlm.nih.gov/geo/query/acc.cgi?acc=GSE183684). An h5ad file, ^4^ which includes cell annotation from detailed molecular characteristics analysis of embryonic retinal cells, was downloaded from the CZ CELLxGENE Discover platform ( https://cellxgene.cziscience.com/collections/c11009b8-b113-4a99-9890-78b2f9df9d79). License: OSI-approved open-license software is under the terms of the Creative Commons Zero “No rights reserved” data waiver (CC0 1.0 Public domain dedication). Archived Additional Tables available from: https://doi.org/10.5281/zenodo.21755650 ^58^ License: OSI-approved open-license software is under the terms of the Creative Commons Zero “No rights reserved” data waiver (CC0 1.0 Universal). The project contains the following underlying data: • Additional_Table_1.csv. • Additional_Table_2.csv. • Additional_Table_3.csv. • Additional_Table_4.csv. • Additional_Table_5.csv. • Additional_Table_6.csv. • Additional_Table_7.csv.

## Software availability

Archived source R code available from: https://doi.org/10.5281/zenodo.21755678 ^59^

License: OSI-approved open-license software is under the terms of the Creative Commons Zero “No rights reserved” data waiver (CC0 1.0 Universal).

The project contains the following underlying data:

• 260320_retina_chromatin_d59_before_merge.R (code No.1)
• 260319_retina_chromatin_d59_d74_d78_merged_No0.R (code No.2)
• 260319_retina_chromatin_d59_d74_d78_merged_No1.R (code No.3)
• 260319_retina_chromatin_d59_d74_d78_merged_No2.R (code No.4)
• 260319_retina_chromatin_d59_d74_d78_merged_No3.R (code No.5)
• Watabe_F1000_ver5_new_No1.R (code No.6)
• Watabe_F1000_ver5_new_No2.R (code No.7)
• Watabe_F1000_ver5_new_No3.R (code No.8)
• Watabe_F1000_ver5_new_No4.R (code No.9)
• Watabe_F1000_ver5_new_No5.R (code No.10)
