# Supplementary material for: Expression status transition of  NOTCH1 accompanies chromatin remodeling in human early retinal progenitor cells
Source: F1000Res. 2026 Apr 17;14:31. Originally published 2025 Jan 6. [Version 4] doi: 10.12688/f1000research.159630.4 (PMC13637476; doi:10.12688/f1000research.159630.4)
Supplement: Peer review history

# Response

**Published:** 2026-08-06

**Authors:** Masaharu Yoshihara

**Affiliations:** University of Tsukuba, Tsukuba, Ibaraki Prefecture, Japan

Dear Dr. Tomohiro Masuda,

 Thank you very much for your time and effort in reviewing our manuscript. We appreciate your constructive comments and have significantly revised the manuscript to address all points, using the annotated single-cell RNA-seq data from Kriukov et al. in Developmental Biology (2025). We are submitting a revised version of this manuscript with this response letter.

 Comments from Dr. Tomohiro Masuda:

 1. I understand the technical difficulties associated with single-cell data analysis, but your results suggest that *PAX6* does not sufficiently explain the regulation of *NOTCH* gene expression. Have you searched for factors other than *VSX2*, *LHX2*, *RAX*, and *PAX6* that could more clearly explain *NOTCH* regulation? Searching beyond these known factors might lead to the discovery of novel factors, thereby increasing the value of this report.

 We corrected cell annotation and found that, in line with previous literature by Furukawa et al. in Neuron (2000) and Irie et al. in Molecular and Cellular Biology (2015), the *NOTCH1* locus contains a RAX binding site that has frequent Tn5 insertions. We revised the Discussion section to fully explain PAX6 expression and this RAX binding site.

 2. You mentioned Blimp1 as a developing cone photoreceptor marker gene, but it is not a cone-specific marker; rather, it is a general photoreceptor marker. *THRB*, as shown in Figure 1D, is a more appropriate marker.

 We adopted cell annotation from Kriukov et al. in Developmental Biology (2025) and transferred the cell labeling to make it more concise.

 3. Is your cluster definition of the "PR/BC precursor" correct? You defined *GADD45G* as the marker, but in Figure 1D, the expression levels of *ONECUT2* and *PAX6*genes are much higher than that of *GADD45G* in this cluster. These two genes are not markers for PRs and BCs.

 We adopted cell annotation from Kriukov et al. in Developmental Biology (2025) and transferred the cell labeling to make it more concise.

 4. Is your cluster definition of the "early RPC" correct? The Monocle3 pseudotime analysis shown in Figure 1C suggests that the early RPCs developed later than RPC1–4 ("Later RPCs" in your definition).

 We adopted cell annotation from Kriukov et al. in Developmental Biology (2025) and transferred the cell labeling to make it more concise.

 Again, thank you very much for your constructive comments. We look forward to hearing from you soon.

 Sincerely,

 Masaharu Yoshihara, M.D., Ph.D.

 Specially Appointed Assistant Professor

 University of Tsukuba, Japan

# Response

**Published:** 2026-08-06

**Authors:** Masaharu Yoshihara

**Affiliations:** University of Tsukuba, Tsukuba, Ibaraki Prefecture, Japan

Dear Dr. Benedikt Obermayer,

 Thank you very much for your time and effort in reviewing our manuscript. We appreciate your constructive comments and have significantly revised the manuscript to address all points, using the annotated single-cell RNA-seq data from Kriukov et al. in Developmental Biology (2025). We are submitting a revised version of this manuscript with this response letter.

 Comments from Dr. Benedikt Obermayer:

 In the new version, the authors have re-done the analysis on data integrated over timepoints, which makes cell type labels easier to compare, even though an actual label transfer from Thomas et al. or Zuo et al. would have helped put their results into the context of the literature. Thomas et al. is now cited, but the findings of that paper are not discussed. In particular, I'm still missing a motivation for their re-analysis of that data, or where this re-analysis leads to new or different results: Notch accessibility and expression was already discussed in the supplementary figures of Thomas et al; the identification of PAX6 binding sites at the NOTCH1/NOTCH3 loci is presented as a key novel finding, but these sites are readily visible in the UCSC browser using the JASPAR predicted TF binding track, and the authors' own 'confirmation' via the browser tracks from Thomas et al. is therefore somewhat circular.

 We agree with you that cell annotation is important and transferred the cell label from a recent paper by Kriukov et al. in Developmental Biology (2025), in which the authors extensively examined molecular characteristics of developing retinal cells. This label transfer made it more concise and led to the identification of a new RAX transcription factor binding site instead of PAX6. We believe it is important to report putative CREs alongside transcription factor expression levels, even if chromatin remodeling is visible in the UCSC browser. In addition, our research focuses on the regulation of Notch signaling, which is why we examined the eye, in which cell differentiation is well characterized. We revised the manuscript so that our motivation in Notch signaling biology is clear, and the link to the ophthalmological finding by Thomas et al. is also clear. For example, we have added the following sentences in the Discussion section:

 Although the accessibility of the *NOTCH1* locus is known to decrease during retinal cell differentiation (Thomas et al.), how this chromatin remodeling affects *NOTCH1* expression, along with candidate *cis*-regulatory elements, is still unknown. In this study, we hypothesized that chromatin remodeling and transcription factor levels are involved and sought possible examples using this dataset.

 Again, thank you very much for your constructive comments. We look forward to hearing from you soon.

 Sincerely,

 Masaharu Yoshihara, M.D., Ph.D.

 Specially Appointed Assistant Professor

 University of Tsukuba, Japan

# Response

**Published:** 2025-09-16

**Authors:** Masaharu Yoshihara

**Affiliations:** University of Tsukuba, Tsukuba, Ibaraki Prefecture, Japan

Dear Dr. Zhongjie Tang

 Thank you so much for your time and efforts in reviewing our manuscript titled “Expression status transition of *NOTCH1* accompanies chromatin remodeling in human early retinal progenitor cells”. We appreciate your constructive comments and have tried to include all the points. Here, we would like to submit a revised version of this manuscript with this response letter. We would like to respond to your comments. Your comments are sequentially numbered and followed by our response in a point-by-point manner.

**Comments from Dr. Zhongjie Tang**

 This study presents a reanalysis of publicly available single-cell multi-omics datasets to explore the relationship between NOTCH1 expression dynamics and chromatin accessibility during early stages of human retinal progenitor cell (RPC) differentiation. The authors apply standard single-cell RNA-seq and ATAC-seq integration methods to a biologically relevant question related to Notch signaling in retinal development. While the technical execution appears sound, the analysis remains largely descriptive and would benefit from further depth. Several issues—particularly concerning data interpretation and figure clarity—should be addressed to improve the overall quality of the manuscript.

**Essential Revisions**

**(1) Clarify the expression hierarchy of NOTCH1–3**

 The manuscript currently implies that NOTCH3 is expressed at levels comparable to NOTCH1 across developmental stages. However, Figures 1D, 2D, and 3D clearly show that NOTCH1 is the dominant isoform, while NOTCH3 is expressed at notably lower levels. The text should be revised to accurately reflect these patterns and avoid overgeneralization.

**Response: **Thank you for your constructive comments. We agree to your comments and have carefully revised the manuscript and ensured that *NOTCH1* showed the highest expression, followed by *NOTCH2* or *NOTCH3*. In the revised manuscript (the Results section for day 59 sample), we have emphasized that, considering the literature (ref. 25), we focused on *NOTCH1* and *NOTCH3*. To avoid overgeneralization, we have carried out analyses to the four NOTCH genes and reached to the conclusion that *NOTCH1* mRNA expression and chromatin accessibility concomitantly changed during RPC differentiation.

**(2)** Discuss the discrepancy between NOTCH2 expression and chromatin accessibility

 Although NOTCH2 is transcriptionally active throughout all three stages, there is little to no signal at its locus in the ATAC-seq data. This discrepancy warrants further discussion. Potential explanations might include technical limitations of ATAC-seq, regulation via distal enhancers, or post-transcriptional mechanisms.

**Response: **Thank you for your constructive comment. This point is also raised by Dr. Mariko Kashiwagi, and we have revised our manuscript as described in comment #2.

**(3) Expand chromatin accessibility analysis beyond the immediate gene body**

 The current analysis is restricted to a narrow window around the NOTCH1 locus (Chr9:136510000–136520000). Expanding the analysis region upstream and downstream (e.g., ±50–100 kb) may help identify putative enhancers or other regulatory elements contributing to the observed expression dynamics.

**Response:** Thank you for your constructive comment. This point is also raised by Dr. Mariko Kashiwagi, and we have revised our manuscript as described in comment #1.

**(4) Enhance figure annotation and clarity**

 The figures, particularly the UMAP and coverage plots, would benefit from clearer labeling. Specifically:

 Annotate key functional regions such as promoters or candidate enhancers in the ATAC-seq plots.

**Response: **Thank you for your constructive comment. We agree to your comment and have clearly indicated the peak regions with arrowheads in the ATAC-seq plots in Figure 2, 4 and 6, along with Additional_file_1, 2 and 3. In addition, the exact chromosome regions of each peak are now indicated in supplementary files (e.g., “ATAC_peaks_NOTCH1_d59.csv”) in our GitHub repository ( https://doi.org/10.5281/zenodo.17084024).

 Ensure cluster labels are consistent across UMAPs and provide legends that identify RPC subtypes.

**Response:** Thank you for your constructive comment. We agree to your comment and have corrected all the cluster labels to ensure the consistency among the samples and to be reader friendly.

 A supplementary table listing marker genes and cell counts per cluster would improve clarity for readers.

**Response:** Thank you for your constructive comment. We agree to your comment and have now provided a supplementary table listing marker genes and cell counts per cluster as “Supplementary_table_count.xlsx” in our GitHub repository ( https://doi.org/10.5281/zenodo.17084024).

**Recommended Revisions**

**(5) Provide biological context for NOTCH3**

 Although NOTCH3 is included in the gene expression analysis, the manuscript does not explain its potential role in retinal development. Including a brief discussion of existing literature—or acknowledging the lack thereof—would better justify its inclusion in the study.

**Response: **Thank you for your constructive comment. We agree to your comment and have analyzed *NOTCH3* as well as *NOTCH1* since *NOTCH3* has been suggested for retinal development (ref. 25). Therefore, we have added this reference paper in this revised manuscript. In addition, the biological significance of *NOTCH3* is added in the Introduction section (“In addition to *Notch1*, *Notch3* expression has also been suggested in the early RPCs, which potentially affects the amount and oscillation of total Notch signaling via its susceptibility to the receptor cleavage and subsequent signal transduction).

**(6) Connect findings with known transcriptional regulators**

 The manuscript mentions transcription factors such as LHX2, PAX6, and RAX, which are known regulators of retinal development. Further exploring how these factors may influence NOTCH1 expression or chromatin accessibility—either through motif analysis or referencing prior enhancer studies—would strengthen the biological interpretation.

**Response:** Thank you for your constructive comment. We agree to your comment and have searched for binding motifs for those transcription factors in chromatin remodeling regions in the Notch loci. We have identified several binding motifs (indicated by arrowheads and numbers in Figure 2, 4 and 6 and Additional_file_1, 2 and 3, supporting the potential biological significance. The exact peak and binding motif information is provided in a supplementary file named “Supplementary_table_motif.xlsx” in our GitHub repository ( https://doi.org/10.5281/zenodo.17084024).

**(7) Summary Recommendation**

 Major revision — The study addresses a relevant topic and is based on a solid dataset, but the analysis remains limited and the interpretation lacks depth. With substantial improvements in analytical scope, biological context, and figure presentation, the manuscript could make a valuable contribution to the field.

**Response:** We sincerely appreciate your thoughtful insights, all of which helped us improve the manuscript significantly.

 The R code used in this revised manuscript is available in our GitHub repository ( https://doi.org/10.5281/zenodo.17084033).

 Again, thank you so much for your constructive comments. We look forward to hearing from you.

 Sincerely,

 Masaharu Yoshihara, M.D., Ph.D.

 Specially Appointed Assistant Professor

 University of Tsukuba, Japan

# Response

**Published:** 2025-09-16

**Authors:** Masaharu Yoshihara

**Affiliations:** University of Tsukuba, Tsukuba, Ibaraki Prefecture, Japan

Dear Dr. Mariko Kashiwagi,

 Thank you so much for your time and efforts in reviewing our manuscript titled “Expression status transition of *NOTCH1* accompanies chromatin remodeling in human early retinal progenitor cells”. We appreciate your constructive comments and have tried to include all the points. Here, we would like to submit a revised version of this manuscript with this response letter. We would like to respond to your comments. Your comments are sequentially numbered and followed by our response in a point-by-point manner.

**Comments:**

 In this manuscript, the authors investigated whether NOTCH family genes (NOTCH1–4) were expressed in retinal progenitor cells (RPCs) and whether changes in chromatin accessibility at promoter regions contributed to their transcriptional activation. To address these questions, they reanalyzed publicly available single-nucleus RNA sequencing (snRNA-seq) and snATAC-seq datasets of the human fetal retina across three developmental stages (GSE183684). Overall, the analytical pipelines are valid and appropriately implemented. However, several concerns regarding the experimental design and the interpretation of the results require further consideration.

**Major Concerns and Comments:**

**(1) Chromatin Accessibility Analysis:**

 During development, enhancer regions play a crucial role in dynamic, lineage-specific gene regulation by modulating chromatin structure and recruiting transcription factors. In contrast, promoter regions generally maintain a more stable configuration following cellular differentiation into a specific lineage. With this in mind, I recommend that the authors expand their chromatin accessibility analysis beyond the promoter and gene body to include the 5' upstream and 3' downstream regions. This broader approach may provide a more comprehensive understanding of how various regulatory elements, including enhancers, contribute to the transcriptional regulation of NOTCH genes.

**Response:** Thank you for your insightful comments. We agree to your comment and have expanded the chromatin accessibility analysis to include 100 kb upstream and downstream of the Notch genes, and we have identified additional chromatin remodeling regions. The results are presented in Figure 2, 4 and 6 ( *NOTCH1* and *NOTCH3* for the three samples) and Additional_file_1, 2 and 3 ( *NOTCH2* and *NOTCH4 *for the three samples). We have drastically revised the text for each sample in the Results section and provided the exact chromosome regions of each peak in supplementary files (e.g., “ATAC_peaks_NOTCH1_d59.csv”) in our GitHub repository ( https://doi.org/10.5281/zenodo.17084024).

**(2) Interpretation of the Results:**

 The authors stated in the text that "NOTCH1-3 were expressed primarily in early RPCs, with NOTCH1 and NOTCH3 being the most prominent genes." However, this claim is not fully supported by the data (Fig. 1D, Additional File 1B; Fig. 2D, Additional File 2B; Fig. 3D, Additional File 3B). A comprehensive interpretation of the three developmental stages suggests that NOTCH1 was the predominant isoform expressed in early RPC clusters, although expression levels varied across clusters and stages. At day 59, the expression hierarchy was NOTCH2 > NOTCH3, whereas at days 74 and 78, NOTCH2 and NOTCH3 exhibited similar expression levels. The authors are advised to interpret these data more carefully.

**Response: **Thank you for your constructive comments. We agree to your comment and clearly have indicated that the early RPCs expressed *NOTCH1*, *NOTCH2* and *NOTCH3* for each sample in the Results section. In particular, *NOTCH1* showed the highest expressions throughout the three samples and we have clearly mentioned that we have focused in *NOTCH1* and *NOTCH3* in this study since *NOTCH3*has been suggested for early RPC differentiation (ref. 25) in the Results section for day 59 sample. Since the early RPCs expressed *NOTCH2* in all the three samples, we have also analyzed chromatin accessibility for *NOTCH2* in addition to *NOTCH1* and *NOTCH3*although we are unable to provide solid conclusions regarding this gene owing to varying findings on its chromatin accessibility, which is clearly mentioned in the Discussion section. Our findings and conclusion that *NOTCH1* mRNA expression and chromatin accessibility concomitantly changed during RPC differentiation, however, are solid in front of the unexplained results on *NOTCH2*.

**(3)** Additionally, although NOTCH2 expression was consistently observed across all three stages (days 59, 74, and 78), the corresponding ATAC peaks were either absent or very low. The manuscript did not provide any discussion of this discrepancy. The authors should address this issue to clarify the relationship between chromatin accessibility and NOTCH2 expression, while also discussing whether this discrepancy may stem from technical limitations or reflect underlying biological factors.

**Response: **Thank you for your constructive comments. We agree to your comment and have revised our manuscript by adding several possible mechanisms such as distal enhancers and a post-transcriptional mechanism (small non-coding regulatory RNAs) (ref. 38) as well as technical limitations in the Discussion section. Although we are unable to fully explain the discrepancy of *NOTCH2* mRNA expression and chromatin accessibility, we are confident to the main conclusion regarding *NOTCH1*.

 Sincerely,

 Masaharu Yoshihara, M.D., Ph.D.

 Specially Appointed Assistant Professor

 University of Tsukuba, Japan
